# Keratinocyte differentiation induces APOBEC3A, 3B, and mitochondrial DNA hypermutation

**DOI:** 10.1038/s41598-018-27930-z

**Published:** 2018-06-27

**Authors:** Kousho Wakae, Tomoaki Nishiyama, Satoru Kondo, Takashi Izuka, Lusheng Que, Cong Chen, Kina Kase, Kouichi Kitamura, Md Mohiuddin, Zhe Wang, Md Monjurul Ahasan, Mitsuhiro Nakamura, Hiroshi Fujiwara, Tomokazu Yoshizaki, Kazuyoshi Hosomochi, Atsushi Tajima, Tomomi Nakahara, Tohru Kiyono, Masamichi Muramatsu

**Affiliations:** 1 Department of Molecular Genetics, Graduate School of Medical Science, Kanazawa University, Kanazawa, Ishikawa 920-8640 Japan; 2 Advanced Science Research Center, Kanazawa University, Kanazawa, Ishikawa 920-8640 Japan; 3 Division of Otorhinolaryngology and Head and Neck Surgery, Kanazawa University, Kanazawa, Ishikawa 920-8640 Japan; 4 Department of Obstetrics and Gynecology, Graduate School of Medical Science, Kanazawa University, Kanazawa, Ishikawa 920-8640 Japan; 5 Division of Medical Oncology, Affiliated Zhongshan Hospital of Dalian University, Dalian, 116001 China; 6 Department of Bioinformatics and Genomics, Graduate School of Advanced Preventive Medical Sciences, Kanazawa University, Kanazawa, Ishikawa 920-8640 Japan; 7 Division of Carcinogenesis and Cancer Prevention, National Cancer Center Research Institute, Tokyo, 104-0045 Japan; 8 Department of Virology II, National Institute of Infectious Diseases, Tokyo, 162-8640 Japan; 9 Department of physiology, Kochi Medical School, Kochi University, Nankoku, Kochi, 783-8505 Japan

## Abstract

Mitochondrial DNA (mtDNA) mutations are found in many types of cancers and suspected to be involved in carcinogenesis, although the mechanism has not been elucidated. In this study, we report that consecutive C-to-T mutations (hypermutations), a unique feature of mutations induced by APOBECs, are found in mtDNA from cervical dysplasia and oropharyngeal cancers. *In vitro*, we found that APOBEC3A (A3A) and 3B (A3B) expression, as well as mtDNA hypermutation, were induced in a cervical dysplastic cell line W12 when cultured in a differentiating condition. The ectopic expression of A3A or A3B was sufficient to hypermutate mtDNA. Fractionation of W12 cell lysates and immunocytochemical analysis revealed that A3A and A3B could be contained in mitochondrion. These results suggest that mtDNA hypermutation is induced upon keratinocyte differentiation, and shed light on its molecular mechanism, which involves A3s. The possible involvement of mtDNA hypermutations in carcinogenesis is also discussed.

## Introduction

Associations have been found between mitochondrial DNA (mtDNA) mutations and cervical and oral squamous cell carcinoma^1–5^. This has been linked with the Warburg effect, a metabolic shift from oxidative phosphorylation to glycolysis in tumour cells^6,7^. Oxidative phosphorylation abundantly produces reactive oxygen species (ROS), and their proximity to mtDNA is believed to play important roles in the accumulation of mtDNA mutations. mtDNA mutations potentially result in dysfunction of the respiratory chain complex, increasing ROS levels, DNA oxidation, genomic instability, and tumourigenesis^6,7^. mtDNA mutations are associated with distant metastases in lung and colon cancers, and are experimentally shown to facilitate the growth and metastasis of cancer cells^8–11^. However, the exact mechanisms for how mtDNA mutations are induced and how they contribute to carcinogenesis remain to be elucidated. The majority of mtDNA mutations are believed to be created via DNA oxidization, especially guanine oxidization into 8-oxoguanine (8-oxoG), which results in G:C to T:A mutations^12^. However, mutations other than G-to-T are also often found^4,13^, suggesting that ROS are not the sole mutagen for mtDNA.

APOBEC proteins are a family of enzymes that convert cytosine into uracil in DNA and RNA. In humans, the APOBEC family is composed of 11 members, including AID and APOBEC1, 2, 3A, 3B, 3C, 3DE, 3F, 3G, 3H, and 4^14–17^. APOBEC3s (A3s) are counteracting factors against viruses such as HIV-1 and transposable elements^14–17^. As well as their established role as antiviral molecules, it has been proposed that they may be oncogenic by hypermutating host genomes. *In vivo*, consecutive C-to-T/G-to-A mutations, which share features with those introduced by APOBECs, are found in multiple human tumours including bladder, cervical, lung, head, and neck, and breast cancers^18,19^. Also, APOBEC3A (A3A) expression is correlated with genomic integration of an oncogenic virus, Human Papillomavirus 16 (HPV16) in oropharyngeal cancer (OPC)^20^. *In vitro*, A3A mutates chromosomal genes *TP53* and *c*-*myc*^20,21^, as well as causes DNA strand breaks^22–24^. Regarding the mitochondrial genome, Suspene *et al*. reported that overexpression of A3A in HeLa cells resulted in mtDNA hypermutation^21^. Although the ability of A3s to hypermutate mtDNA has been demonstrated, the pathophysiological context for this remains to be determined.

We hypothesized that A3s induce mtDNA mutation, contributing to carcinogenesis. In this study, we have attempted to clarify the molecular mechanism underlying how this may occur.

## Results

### Hypermutated mtDNA in cervical dysplasia and oropharyngeal cancer

**Figure 1:**
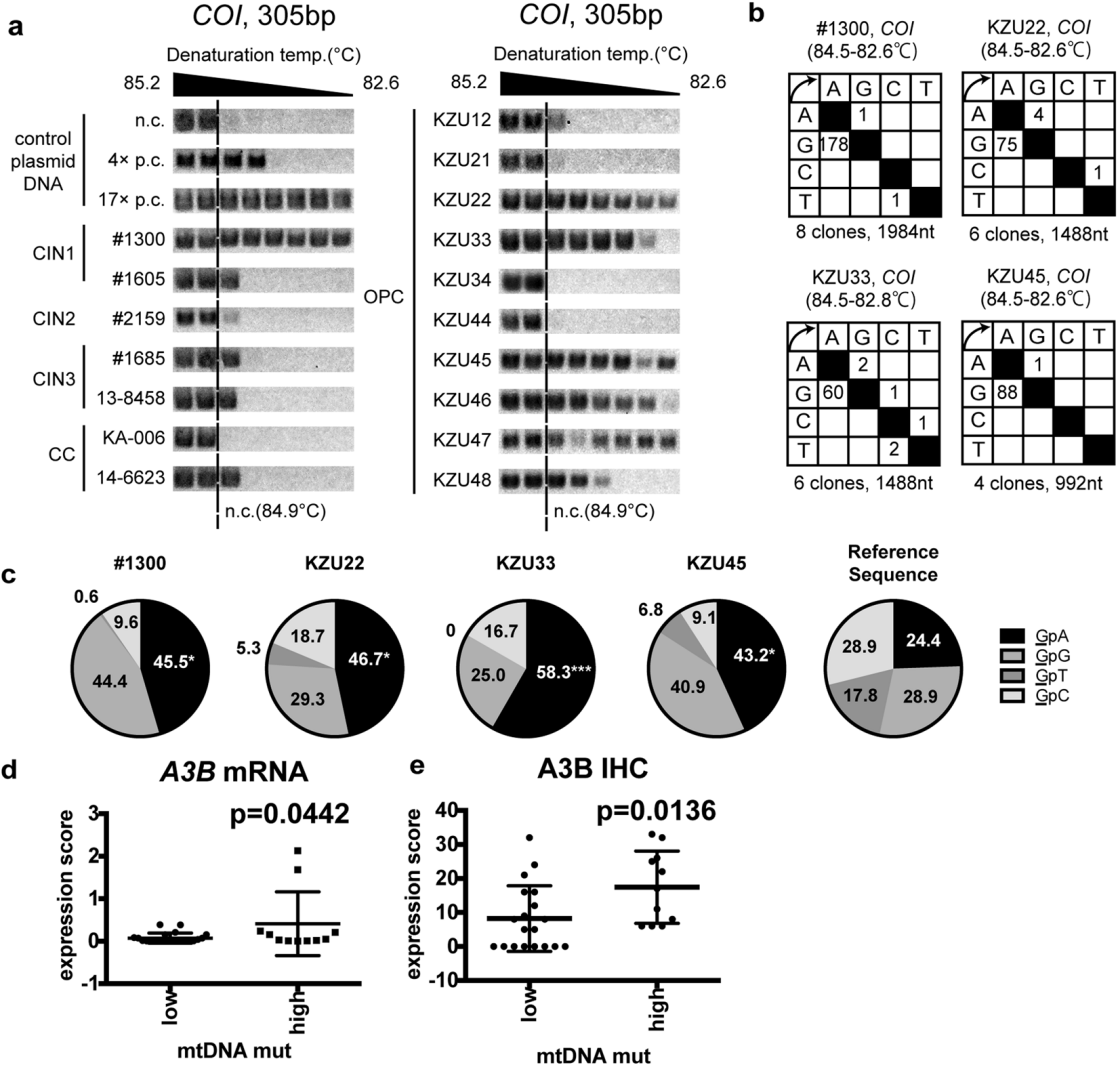
mtDNA hypermutation in cervical dysplasia and oropharyngeal cancer. Total DNA samples were extracted from cervical intraepithelial neoplasia (CIN) 1/2 vaginal swabs, paraffin-embedded HPV16 CIN3, cervical cancer, and oropharyngeal cancer (OPC). (**a**) 3D-PCR analysis targeting *COI* was performed along with plasmids containing *COI* amplicons with the indicated number of C-to-T mutations as control reactions (n.c. indicates the no-mutation control, whereas 4× and 17× indicate 4 and 17 C-to-T mutations, respectively). The dotted lines indicate the lowest denaturing temperature, 84.9 °C, at which *COI* was amplified from n.c. The results for seven cervical dysplasia/cancer and ten OPC samples are shown representatively. (**b**,**c**) 3D-PCR products amplified at denaturation temperatures of 84.5 °C or lower were sequenced and mapped to a reference sequence (GenBank accession number: NC_012920.1). The identified mutations are summarized in matrices (**b**) and the result of the 5′-dinucleotide context analysis is shown in (**c**) (*P < 0.05 and ***P < 0.005). (**d**,**e**) RT-qPCR (**d**) and immunohistochemical analysis (**e**) of A3B in OPCs. OPC samples were divided into two groups according to the lowest denaturing temperature at which *COI* was amplified (mtDNA mut low; >84.1 °C, high; <=84.1 °C). (**e**) A3B protein levels were indicated as expression scores, based on the frequency of immunoreactive cells.

It has been reported that mtDNA was mutated in many types of cancers, including cervical cancer (CC) and OPC^13,25,26^. We have previously reported that A3s are abundantly expressed in the human cervix, OPCs, and cervical intraepithelial neoplasia (CIN) or OPC cell lines^20,27,28^. In addition, HPV16 *E6* and *E7* reportedly induce A3A and APOBEC3B(A3B) expression^29,30^. These findings led us to hypothesize that A3s hypermutate mtDNAs in HPV16-related dysplasia and cancer, and to analyse hypermutations in mtDNA with high sensitivity by using differential DNA denaturation PCR (3D-PCR)^21^. Total DNAs from 10 cervical specimens (two CIN1, one CIN2, five CIN3, and two CC) were subjected to 3D-PCR targeting *COI* and *ND2*, along with the control plasmid DNA containing 0, 4, and 17 C-to-T mutations for *COI*, and 0, 9, and 14 for *ND2*, respectively. For the *COI* control plasmids, the lowest temperatures of amplification were 84.9 °C, 84.1 °C, and 82.6 °C for 0, 4, and 17 C-to-T mutations, respectively (Fig. 1a). For the *ND2* control plasmids, the lowest temperatures were 83.5 °C, 82.6 °C, and 81.9 °C for 0, 9, and 14 C-to-T mutations, respectively (Supplementary Fig. S1a). These indicate that our 3D-PCR analysis was able to quantify mutation load per amplicon.

*COI* was amplified from 8 out of 10 samples at a denaturing temperature lower than 84.9 °C, the lowest at which *COI* is amplified from no-mutation control plasmids (Fig. 1a). Notably, it was amplified at a temperature as low as 82.6 °C for one of the CIN1 specimens (#1300). *ND2* was also amplified from 3 out of 10 samples at temperature lower than 83.5 °C, the lowest at which *ND2* is amplified from no-mutation control plasmids (Supplementary Fig. S1a). It could be amplified at temperatures of 81.9 °C, 82.2 °C, and 82.6 °C from specimens #1300, #2159, and KA-006, respectively. In addition, 33 OPC specimens were tested for *COI* 3D-PCR and it was found that *COI* could be amplified from 11 samples at denaturing temperature at 84.5 °C or less (Figs 1a and S2a). The PCR products amplified at denaturing temperatures lower than 84.9° (*COI*) or 83.5 °C (*ND2*) were collected from four samples for *COI* (#1300, KZU22, KZU33, and KZU45) and two for *ND2* (#1300 and KA006), and sequenced. The result revealed accumulated G-to-A mutations, but no other types of mutation, a unique feature of APOBEC-mediated hypermutation (Figs 1b and S1b). The presence of exclusive G-to-A mutations in these samples suggests that A3s target cytosine in the complementary strand. Dinucleotide preference analysis of C-to-T/G-to-A mutations revealed a bias toward TpC and CpC, over ApC or GpC (Figs 1c and S1c), consistent with the reported characteristics for A3s^31–34^. These results indicate that mtDNA is hypermutated in CIN and OPC samples.

Because HPV16 reportedly induces A3A and A3B^29,30^, the association between HPV16 infection and mtDNA hypermutation was examined. When the threshold was set at 84.5 °C (4 × p.c.), 33% of both HPV16 (+) and HPV (−) OPCs were positive for mtDNA hypermutation, suggesting no obvious association between infection and hypermutation (Supplementary Fig. S2b). With regards to the correlation between A3 expression and mtDNA hypermutation, we found that both mRNA and protein levels of A3B were significantly higher in specimens with mtDNA hypermutation than those without hypermutation (Fig. 1d and e). For other A3s, we could not find obvious correlation between their expression and the load of mtDNA mutations (Supplementary Fig. S3).

We further investigated whether chromosomal and HPV16 DNA hypermutations were associated with mtDNA hypermutation. We used 3D-PCR to target *TP53*, but we were unable to find any sign of hypermutation greater than four C-to-T plasmid controls (Supplementary Fig. S4a). For HPV16 *E2*, we found that one out of two OPCs and one CIN2 contained hypermutation (Supplementary Fig. S4b), as we previously reported^28^. Although it is possible that hypermutation of mtDNA is associated with hypermutation of HPV16 DNA, we were unable to draw firm conclusion from the available samples in this study.

### Frequency of hypermutated mtDNA

To determine the frequency of hypermutated mtDNA in the clinical samples, mtDNA from two CIN (#1300 and #2159) and two OPC samples (KZU33 and KZU45) were sequenced by next-generation sequencing (NGS). In practice, the whole mitochondrial genome, covered by four PCR amplicons, was amplified by conventional PCR (Supplementary Table), along with a plasmid containing D-loop, rRNA, and *ND1* genes (nt16331-nt3729, NC_012920.1), to assess the frequency of sequencing errors related to NGS procedures, including PCR, library construction, and data analysis. We obtained approximately 1–2 million reads from each amplicon mapped to the mitochondrial genome (deposited in DRA, accession numbers. DRA005375 and DRA005377 for CIN and OPC samples, respectively). Mutations were determined by comparison between output reads and a consensus sequence built from each amplicon. The reads were sorted according to the number of C-to-T/G-to-A mutations per read (Supplementary Fig. S5). As we previously reported^28^, the number of reads in the plasmid control decreased exponentially with the number of mutations per read, and we did not detect any with four or more mutations (Table 1). Thus, most reads with four or more mutations are likely to represent hypermutation *in vivo*, rather than the coincidence of multiple PCR or sequencing errors.

**Table 1:** Hypermutated mtDNA in CINs and OPCs (D-loop-*ND1*).

D-loop - *ND1*		number of C-to-T or G-to-A per read							
		0 to 3	4	5	6	7	8	10	22
	plasmid	2423061	0	0	0	0	0	0	0
CIN1	#1300	2382107	8	8	4	2	0	1	0
CIN2	#2159	2121943	0	0	0	0	0	0	0
OPC	KZU33	1303978	0	0	0	0	0	0	0
OPC	KZU45	1150214	2	0	1	0	1	0	1

**Figure 2:**
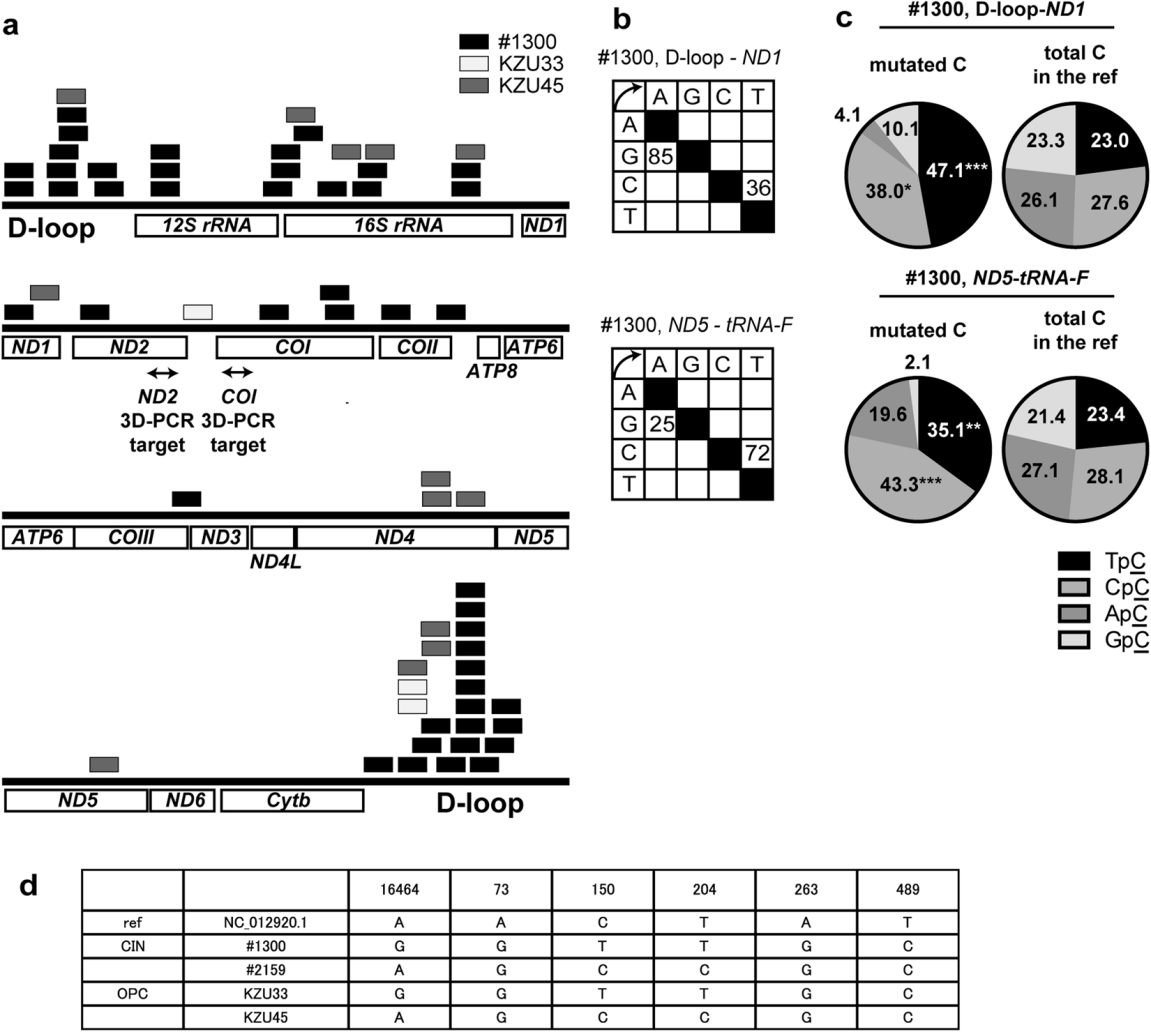
Distribution and frequency of mtDNA hypermutation. D-loop–*ND1* (nt16331–nt3729, NC012920.1), *ND1*–*ATP6* (nt3646–nt9158, NC012920.1), *ATP6*–*ND5* (nt8753–nt12831, NC012920.1), and *ND5*–*tRNA*-*F* (nt12540–nt645, NC012920.1) were amplified from two cervical intraepithelial neoplasia (CIN) and two oropharyngeal cancer samples along with a plasmid without mutation. The PCR products were subjected to next-generation sequencing and the output reads were sorted according to the number of C-to-T or G-to-A mutations per read (Tables 1–4). Those with four or more C-to-T/G-to-A mutations were extracted. (**a**) Alignment of reads with four or more C-to-T/G-to-A mutations. The rectangles indicate the positions of the extracted reads in the reference sequence of each sample. (**b**,**c**) Mutation matrices (**b**) and dinucleotide analysis (**c**) of the hypermutated reads, obtained from D-loop–*ND1* and *ND5*–*tRNA*-*F*, amplified from CIN sample #1300. In (**c**), the contexts of Cs in the reference sequences that corresponded to the mutated Cs in the output reads were also analyzed to calculate the expected frequency of each context. The P-values were calculated using the chi-square test, comparing the numbers of TpC and non-TpCs, or CpC and non-CpCs. **P < 0.01 and ***P < 0.005. (**d**) The consensus sequences obtained from #1300, #2159, KZU33, and KZU45 were aligned with revised Cambridge Reference Sequence (NC_012920.1), and the discordant bases were summarized.

Of the analyzed samples, #1300, KZU33, and KZU45 but not #2159 contained hypermutated mtDNA, as summarized in Tables 1–4 and Fig. 2a. Its frequency was highest in the D-loop–*ND1* region of #1300, although it was estimated to be as low as 9.66 ppm (=23/2382130, Table 1). Hypermutated reads from the D-loop–*ND1* and *ND5*–*tRNA*-*F* regions exclusively contained C-to-T/G-to-A mutations, with no other types (Fig. 2b). Both C-to-T and G-to-A rich reads appeared to be concentrated on D-loop from #1300 (Figs 2a and S6). Dinucleotide analysis further revealed a significant TpC and CpC bias (Fig. 2c), consistent with the proposed signature of A3-mediated hypermutation^31,32^.

**Table 2:** Hypermutated mtDNA in CINs and OPCs (*ND1*-*ATP6*).

*ND1* - *ATP6*		number of C-to-T or G-to-A per read					
		0 to 3	4	5	6	7	22
CIN1	#1300	2817768	4	1	0	2	0
CIN2	#2159	2443256	0	0	0	0	0
OPC	KZU33	2015183	1	0	0	0	0
OPC	KZU45	1729775	0	0	0	0	1

**Table 3:** Hypermutated mtDNA in CINs and OPCs (*ATP6*-*ND5*).

*ATP6* - *ND5*		number of C-to-T or G-to-A per read			
		0 to 3	5	10	13
CIN1	#1300	2093340	1	0	0
CIN2	#2159	1774190	0	0	0
OPC	KZU33	1298772	0	0	0
OPC	KZU45	1234401	0	1	2

**Table 4:** Hypermutated mtDNA in CINs and OPCs (*ND5*-*tRNA*-*F*).

*ND5* - *tRNA*-*F*		number of C-to-T or G-to-A per read					
		0 to 3	4	5	6	10	12
CIN1	#1300	2419905	8	4	4	1	1
CIN2	#2159	2175074	0	0	0	0	0
OPC	KZU33	1358320	1	1	0	0	0
OPC	KZU45	1209281	2	0	1	0	1

Since the mitochondrial genome, especially in the D-loop, was frequently found to be mutated in cervical and oral cancers^1–5^, we built the consensus sequences of the D-loop from the libraries and aligned these with the revised Cambridge reference sequence (rCRS, NC_012920.1). We identified mismatched bases, including A16464G, A73G, C150T, T204C, A263G, and T489C (Fig. 2d). Of note, C150T has been reportedly associated with increased risk of cervical cancer^3^.

Overall, these data indicate that mtDNA hypermutation occurred in most of regions in mitochondrial genome with some biased distribution, albeit at a lower frequency compared to that of HPV16 viral hypermutation^28^.

### A3A, A3B, and mtDNA hypermutation induced by keratinocyte differentiation

**Figure 3:**
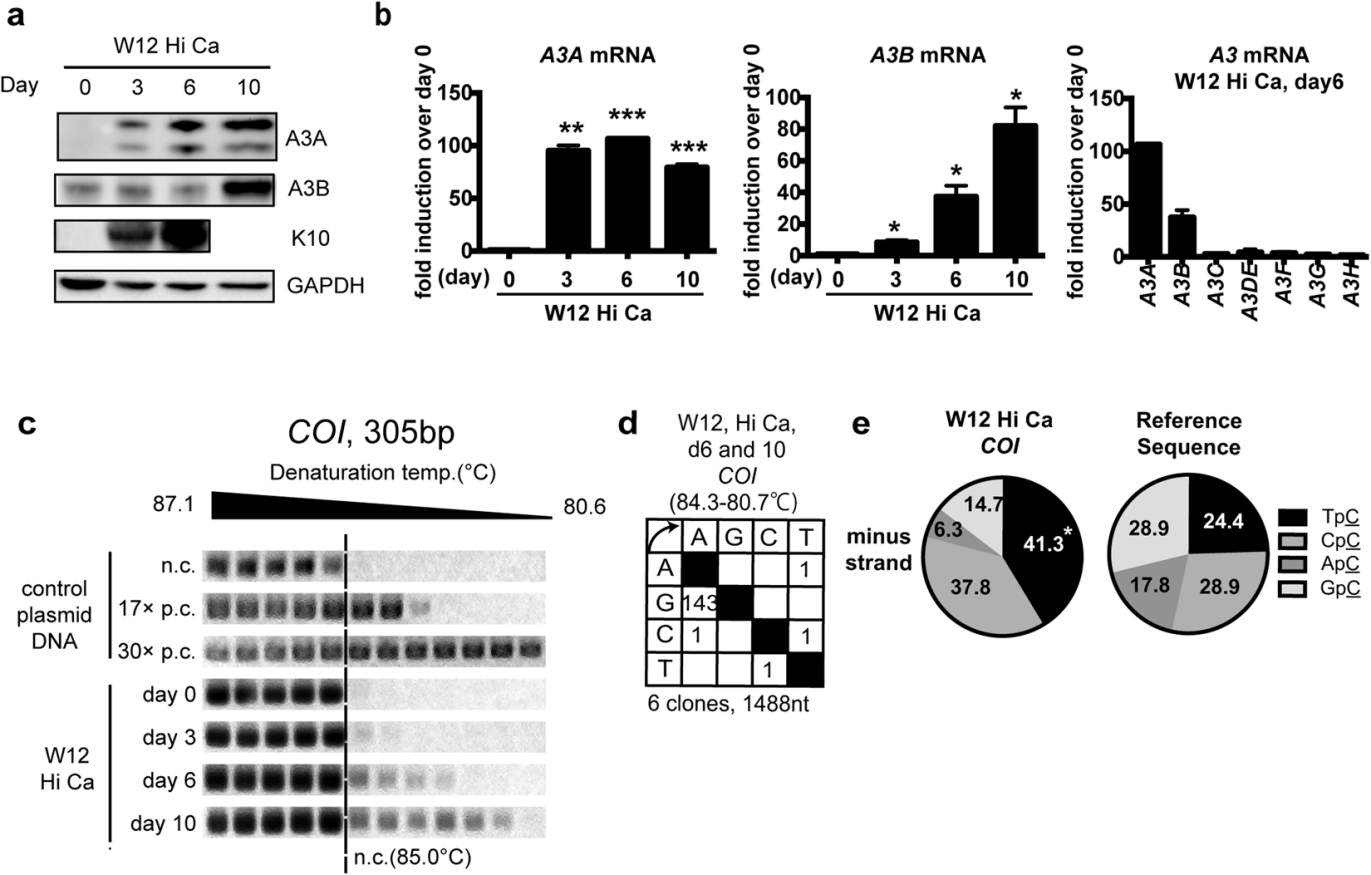
Keratinocyte differentiation induced A3A/B expression and mitochondrial DNA hypermutation. W12 cells were differentiated in 1.2 mM CaCl_2_ for the indicated period of up to 10 days. (**a**) Nine micrograms of total cell lysate were subjected to immunoblotting, to determine the protein levels of A3s, Keratin 10 (K10), and GAPDH. (**b**) RT-qPCR analysis of differentiated W12 cells. Total RNA was harvested and mRNA levels of *A3s* were quantified by RT-qPCR. The copy numbers of *A3s* were normalized to that of *HPRT* and indicated as fold induction relative to day 0. The mean values and the standard deviations are indicated as bar graphs and error bars, respectively. (**c**) Total DNAs were subjected to 3D-PCR targeting *COI*, along with plasmid controls containing amplicons with 0, 17, and 30 C-to-T mutations. (**d**,**e**) The amplicons from a denaturing temperature of 85.0 °C or lower were sequenced (**d**), and the dinucleotide preference of G-to-A mutations are summarized (**e**). The P-values were calculated using unpaired two-tailed Student’s t tests and chi-square tests for the RT-qPCR analysis and dinucleotide analysis, respectively. *P < 0.05, **P < 0.01, and ***P < 0.005.

To investigate the mechanism for mtDNA hypermutation found in CIN and OPC, we made use of a keratinocyte cell line, W12 cells, established from CIN1, which differentiates into keratinocytes under a high extracellular calcium concentration^35,36^. By cultivation up to day 10, we confirmed upregulation of an early differentiation marker, K10, by western blotting (Fig. 3a). In this experimental condition, the expression of *A3s* was profiled and the results indicated 79.25 and 82.05-fold induction of *A3A* and *A3B* at day 10 compared with day 0, respectively (Fig. 3b). The levels of other *A3s* had changed by less than five-fold at day 6 (Fig. 3b). Increased protein levels of A3A and A3B were also confirmed by western blotting (Fig. 3a). Total DNA was subjected to 3D-PCR analysis that targeted *COI*. As expected, *COI* was amplified at a denaturing temperature lower than 85.0 °C (that of the no-mutation control, Fig. 3c) by culturing cells in a differentiating condition beyond day 6 (Fig. 3c). Sequencing the amplicons revealed an obvious bias of G-to-A over C-to-T mutations, suggesting that A3s target cytosine in the non-coding strand of *COI* gene (Fig. 3d), with a dinucleotide bias toward TpC and CpC (Fig. 3e), as in clinical samples. We also found that *TP53* was not hypermutated in this condition (Supplementary Fig. S7).

To examine whether differentiation-induced mtDNA hypermutation was dependent on HPV, we also examined HPV (−)  keratinocytes. Human epidermal keratinocytes immortalized by the ectopic expression of TERT, a mutant form of CDK4 and cyclin D1 (HDK-K4DT)^37^, were induced to differentiate, and *A3* profiling and mtDNA 3D-PCR analysis were performed. The results indicated that *A3A* (but not *A3B*) mRNA was upregulated 15-fold by 72 h (Supplementary Fig. S8a), and western blotting confirmed an increased protein level of A3A as well as the differentiation marker K10 (Supplementary Fig. S8b). It has been reported that A3A consists of two isoforms^24^; the shorter isoform p2 was undetectable in this cell line. Under this condition, the results from *COI* 3D-PCR indicated an increased mutation load in differentiated HDK-K4DT compared to undifferentiated HDK-K4DT (Supplementary Fig. S8c). The mutation was biased to non-coding strand, as in W12 (Supplementary Fig. S8d).

For further verification, the differentiation of normal human epidermal keratinocytes (NHEK) was induced; the mRNA and protein levels of A3B, and mtDNA hypermutations were found to be increased (Supplementary Fig. S8e–g). Taken together, these findings suggest that keratinocyte differentiation induced A3 expression and mtDNA hypermutation concurrently in an HPV-independent manner.

### Role of A3A/A3B in mtDNA hypermutation

**Figure 4:**
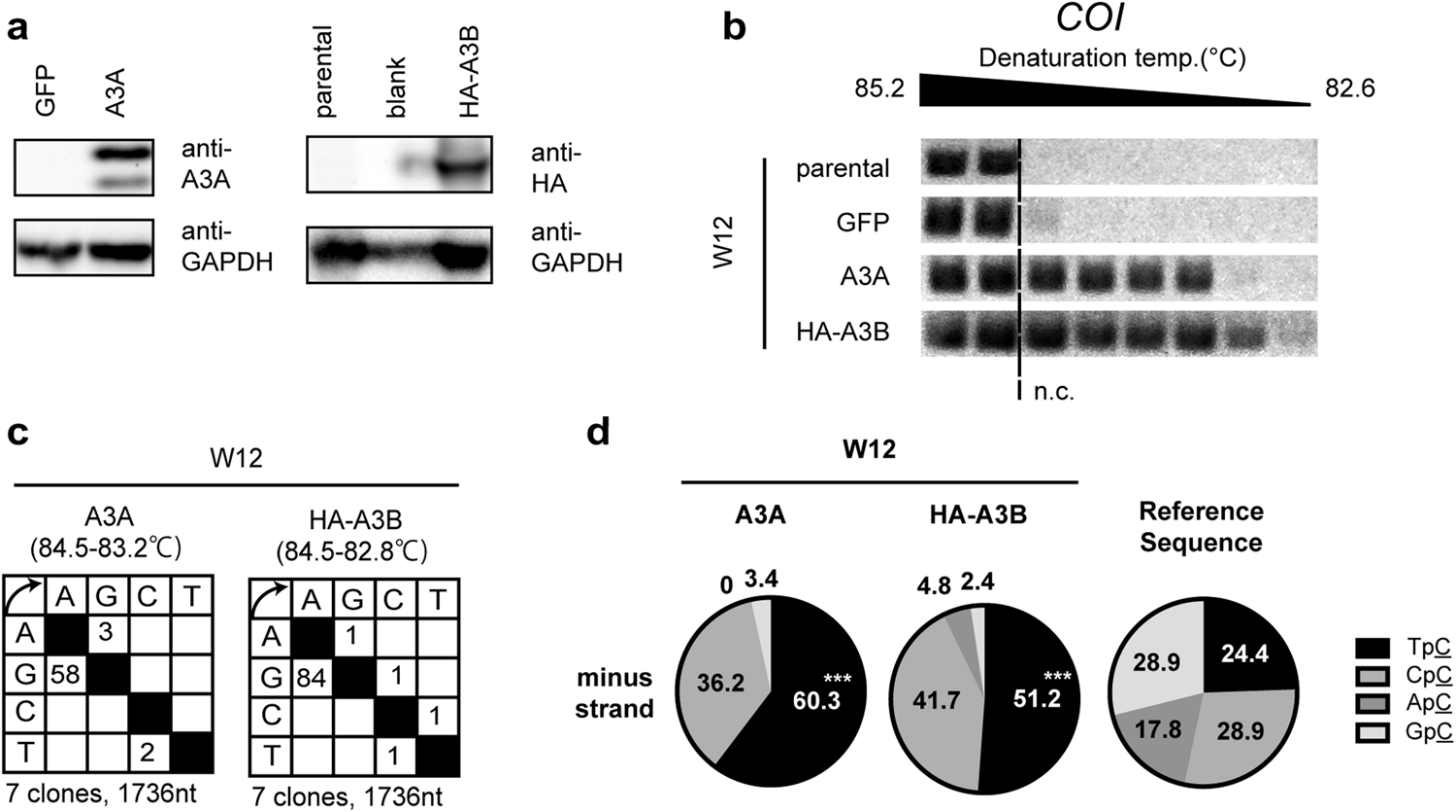
A3A and A3B hypermutated mitochondrial DNA. W12 cells were transfected with expression vectors for GFP, A3A, and HA-A3B, and cultivated in maintenance medium for 48 h. (**a**) The ectopic expression of transgenes was validated by western blotting. GAPDH served as a loading control. (**b**) Total DNAs were subjected to 3D-PCR analysis targeting *COI*. (**c**,**d**) PCR products amplified at a denaturing temperature of 84.5 °C or lower were sequenced (**c**); the dinucleotide preferences of G-to-A mutations are summarized (**d**). The P-values were calculated by chi-square test. ***P < 0.005.

To investigate whether the exogenous expression of A3A or A3B (the two major responders among A3s to high-calcium culture in W12 cells) hypermutated mtDNA, these A3s were ectopically expressed in W12 cells (Fig. 4a). 3D-PCR analysis targeting *COI* revealed that A3A and A3B expression increased the mutation load compared to a control GFP transfectant (Fig. 4b). Sequencing the PCR product amplified at a denaturing temperature lower than 84.9 °C (that of the no-mutation control) revealed accumulated mutations on the non-coding strand in the A3A and A3B overexpressed W12 cells (Fig. 4c), and the biased dinucleotide preference toward TpC (Fig. 4d), as in the CINs, OPCs, and differentiated W12 cells (Figs 1 and 3).

To further verify whether endogenous A3A or A3B is involved in mtDNA hypermutation in differentiated W12 cells, we transfected siRNA against A3A. Unexpectedly however, siRNA transfection decreased mtDNA hypermutation (Supplementary Fig. S9a and b), hampering us from assessing the contribution of endogenous A3s. In addition, we lentivirally transduced shRNA against A3A or A3B, and found that mtDNA hypermutation load was comparable between shA3A, shA3B, and control shRNA infectants (Supplementary Fig. S9c–e), implying that induction of either A3A or A3B was sufficient for reducing mtDNA hypermutation.

Since ROS has been demonstrated to regulate keratinocyte differentiation^38,39^, we attempted to verify its involvement in A3A/B induction and mtDNA hypermutation in differentiated W12 cells. To this end, W12 cells were induced to differentiate in the presence of a ROS-antagonist, Mito-Q. Results showed that mRNA levels of differentiation markers, involucrin and loricrin, were decreased in the presence of 1 μM or 2 μM Mito-Q as compared with those of vehicle control (Supplementary Fig. S10). In this condition, A3A was parallelly decreased both at the mRNA and protein level. In contrast, the amount of A3B mRNA, but not that of its protein was obviously affected. Moreover, mtDNA hypermutation was comparable among vehicle and Mito-Q-treated cells. The result did not provide evidence that ROS contributed to mtDNA hypermutation.

### A3A and A3B are contained in the mitochondrial fraction

**Figure 5:**
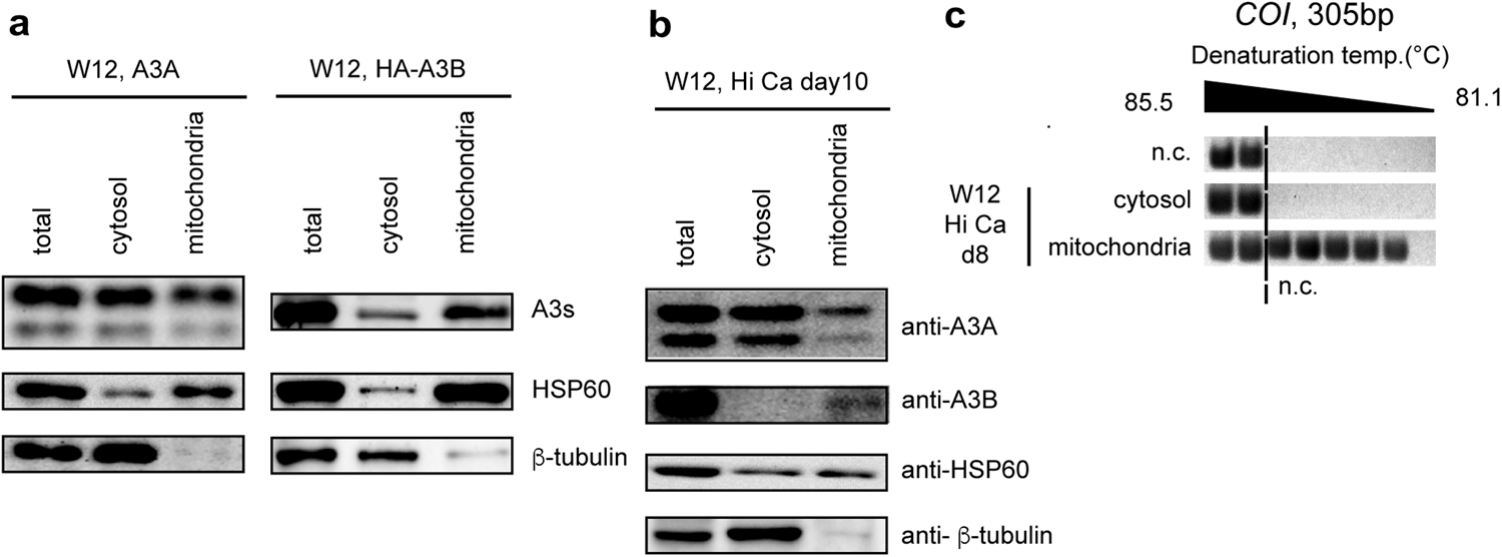
A3A and A3B were contained in the mitochondria. (**a**) W12 cells were transfected with an expression vector coding A3A or HA-A3B, and cultivated in maintenance medium for 48 h. Their lysates were fractionated into cytosolic and mitochondrial fractions, and subjected to western blotting analysis. (**b**,**c**) W12 cells were induced to differentiate under high extracellular calcium concentration, and fractionated into cytosol and mitochondrial fraction, (**b**) Each fraction was subjected to immunoblotting of A3s, HSP60 (a mitochondrial marker), and β-tubulin. (**c**) DNA was extracted from each fraction and subjected to 3D-PCR analysis targeting *COI*.

To examine whether APOBECs could be distributed in mitochondria, nucleus-free lysates from proliferating W12 cells that overexpressed A3A or HA-tagged A3B were fractionated into mitochondrial and non-mitochondrial cytosolic fractions. Immunoblotting revealed that the mitochondrial fraction contained both A3A and A3B (Fig. 5a). Differentiated W12 cells were also fractionated and subjected to immunoblotting; although a fair amount of A3A and A3B remained in the nuclear (and unlysed) fraction (Supplementary Fig. S11), as reported in^24,40^, endogenous A3A and A3B were consistently detected in the mitochondrial fractions(Fig. 5b). DNA fragments were separately extracted, and subsequent *COI* 3D-PCR revealed that the mitochondrial fraction contained hypermutated mtDNA (Fig. 5c). Overall, these results suggested that A3A and A3B could potentially be contained in the mitochondria.

## Discussion

In this study, we demonstrated using 3D-PCR and NGS that cells from CIN and OPC specimens contained hypermutated mtDNA, that correlated with A3B expression (Figs 1 and 2, and Supplementary Figs S1 and S2). Using an *in vitro* experimental system for HPV (+) and (−) keratinocytes, we also demonstrated that differentiation concurrently induced A3 expression and mtDNA hypermutation (Figs 3 and S8), regardless of HPV. Overexpression of A3A and A3B resulted in mtDNA hypermutation (Fig. 4). These results provide evidence of the involvement of A3s in differentiation-induced mtDNA mutation.

Cervical tumours have been demonstrated to contain mtDNA mutations, often C-to-T or G-to-A^3,13,41^. It was believed that mtDNA mutations by ROS resulted from DNA oxidization, but we have demonstrated that mtDNA contains copies almost entirely without mutations other than C-to-T/G-to-A (Figs 1–4, and Supplementary Figs S1 and S8). This can only be explained by A3-mediated hypermutation, rather than by direct DNA oxidation, which reportedly generates 8-oxoG resulting in G:C to T:A mutations^42^.

We compared a standard D-loop sequence with consensus sequences obtained from these specimens and identified several mismatches, including C150T, which is reportedly associated with an increased risk of cervical cancer (Fig. 2d). Because of the lack of available paired non-tumour samples, we could not determine whether they are germline or somatic mutations. Assuming that they are somatic mutations, the result is consistent with the reports claiming that CIN contains a mutated D-loop^3^. We demonstrated that the differentiation of not only neoplastic but also normal keratinocytes induces mtDNA hypermutation (Supplementary Fig. S8). Considering that normal differentiated keratinocytes exfoliate, while neoplastic cells do not, it is intriguing to speculate that neoplastic transformation allows accumulation of mtDNA mutations, which are induced by differentiation.

While the ectopic expression of A3A or A3B was sufficient to induce mtDNA hypermutation (Fig. 4), we could not demonstrate the contribution of endogenous A3A or A3B (Supplementary Fig. S9). In addition, Mito-Q treatment did not decrease mtDNA hypermutation, when a fair amount of A3B, but not A3A remained to express (Supplementary Fig. S10). These results suggest that either A3A or A3B induction is sufficient for mtDNA hypermutation.

While both A3A and A3B were upregulated by differentiation of W12 cells (Fig. 3), either A3A or A3B was upregulated in that of HDK-K4DT, or NHEK, respectively (Supplementary Fig. S8). This can be attributed to their origin, cervical dysplastic cells for W12 cells, and normal keratinocytes for others. Also, one can speculate that A3A/A3B induction in response to differentiation differs individually, explaining the differential induction of A3A and A3B in HDK-K4DT and NHEK, respectively. Further studies are warranted to clarify how individual difference and/or dysplastic transformation affect A3A/A3B induction by differentiation.

We previously reported that A3A expression level correlates with HPV infection in OPCs^20^. It was also reported that HPV16 E6 and E7 reportedly unregulated A3A and A3B^29,30^. In contrast, HPV16 infection was not obviously associated with mtDNA hypermutation (Supplementary Fig. S2), and the differentiation of HPV (−) keratinocytes also hypermutated mtDNA (Supplementary Fig. S8). Nonetheless, overexpression of A3A or A3B was sufficient to hypermutate mtDNA (Fig. 4). This discrepancy can be explained as follows; A3 expression determines mtDNA mutation load, and HPV16 alone cannot induce the expression level of A3s enough for exceeding the threshold for mtDNA hypermutation. Besides, we do not deny possible involvement of negative regulators associated with HPV16.

mtDNA hypermutation correlated with A3B, but not clearly with A3A (Figs 1d,e and S3). A3A is abundantly expressed in hematopoietic cells, especially, macrophages^32,43^, and as mentioned above, HPV16 E7 induces A3A expression^30^. Thus, we speculate that contamination of blood cells, HPV infection, and other unidentified factors may have masked the correlation between A3A and mtDNA hypermutation *in vivo*. Further studies are warranted to verify whether factors other than differentiation affect mtDNA hypermutation.

Suspene *et al*. demonstrated that ectopic A3A was able to hypermutate mtDNA^21^. Consistent with this, we demonstrated that A3A induction and mtDNA hypermutation occurred concomitantly in differentiating keratinocytes (Figs 3 and S8), and that the ectopic expression of A3A was sufficient to induce mtDNA hypermutation in undifferentiated W12 cells (Fig. 4). They claimed that hypermutated mtDNA was confined to the cytosol, based on their data that hypermutated mtDNA was vulnerable to treatment with Triton X-100 and DNase I. Indeed, we found that the mitochondrial fraction of differentiated W12 cells contained hypermutated mtDNA (Fig. 5c). Given that A3A and A3B are contained in the mitochondrial fraction (Fig. 5), and that the cervical cancer mitochondrial genome contains substantial C-to-T/G-to-A mutations, we favour the interpretation that A3s can enter the mitochondria and mutate mtDNA *in situ*. Nevertheless, there remains a possibility that hypermutated mtDNA is also contained in lysosomes, where damaged mitochondrion are degraded by mitophagy^44^, and whose content will inevitably contaminate the mitochondrial fraction. Further investigation is warranted to verify that mtDNA is mutated by APOBECs *in situ* and inherited by daughter cells.

We found by 3D-PCR targeting *COI* that hypermutation accumulated on the complementary, but hardly the coding strand (Figs 1, 3 and 4, and Supplementary Figs S1 and S8). Also, NGS analysis revealed that the mtDNA from specimen #1300 contained significant hypermutation on the D-loop (Figs 2 and S6), which contains the replication origin^45,46^. These findings are consistent with a report by Ju *et al*.^47^, that somatic C-to-T substitution was biased to the strand from which most mitochondrial genes are transcribed, and that it was exceptionally distributed in both strands of D-loop. APOBECs take single-stranded DNA and RNA as their substrates and the hypermutation by A3s follows replication and/or transcription^48^. Thus, these findings may imply that mtDNA hypermutation is coupled to replication.

In summary, we have demonstrated that *in vivo*, mtDNA was hypermutated in CIN, CC, and OPC, and that, *in vitro*, keratinocyte differentiation induced mtDNA hypermutation and A3s expression. mtDNA mutations lead to decreased mitochondrial function, and increased levels of ROS^8,10^. ROS induce mutations, alter the property of cancer cells such as growth and metastasis^8–10^, and promote HPV integration^49^. We had previously proposed that A3s causes the HPV genome to mutate, and promotes its integration^20,27,28^. Other studies have proposed that A3s mutate the chromosomal genome and invoke DNA damage response^21,22,50,51^. Thus, it is intriguing to speculate that A3s and ROS act in synergy to accumulate mutations in the chromosomal, mitochondrial, and HPV genome, as well as to promote HPV integration and chromosomal damage (Supplementary Fig. S12). Further studies are required to understand the role of A3s, mtDNA hypermutation, and ROS in tumourigenesis.

## Materials and Methods

### Clinical samples

Cervical swabs and tumour specimens were collected from patients positive for HPV16 and diagnosed with CIN, CC, or OPC at the Department of Obstetrics and Gynecology or Otorhinolaryngology, Kanazawa University Hospital, Japan. The clinical diagnosis was made by histological analysis of punch biopsy samples obtained under colposcopy for CIN and during surgery for CC and OPC. HPV16 was detected from cervical swabs or oropharyngeal specimens as described in^28^ and^20^, respectively. For CC specimens, it was determined by PCR amplifying HPV16 *E6E7*, as described in^52^ (Supplementary Table). Total DNA was isolated using the QIAamp DNA Mini Kit (Qiagen, Hilden, Germany) for cervical swabs and OPC surgical specimens, or TaKaRa DEXPAT Easy (Kusatsu, Japan) for CC surgical specimens. All the samples were collected after obtaining written informed consent from the patients and were used with the approval of the Ethics Committee of Kanazawa University. This study was performed in accordance with the World Medical Association’s Declaration of Helsinki.

### 3D-PCR analysis

Total DNAs were extracted from the cells as described in^20,27^.For 3D-PCR targeting *COI*, *ND2*, and *TP53*, we followed the protocol as described in^21^, and HPV16 *E2* in^20,27,28,53^. The control plasmids were prepared by cloning PCR products containing the indicated number of C-to-T mutations into pGEM-T Easy Vector (Promega, Madison, Wisconsin). All of the plasmids used in this study were sequenced at least once for verification. To identify mutations, GenBank accession number NC_012920.1 was used as a reference sequence for human mtDNA.

### RT-qPCR

The synthesis of cDNA and qPCR were performed as previously reported^27,28,54–57^. The primers used are listed in Supplementary Table. To determine copy numbers of *A3*s *and HPRT1*, standard curves were generated to quantify the number of transcripts of each gene. For *INV* and *LOR*, the delta-delta Ct method was adopted, and the results were normalized to *HPRT1*.

### Immunohistochemical analyses

Sections were prepared and stained as described^20,58^. For staining of A3B, the anti-A3B antibody clone ab184990 (1:100, Abcam, Cambridge, UK) was used. Stained sections were evaluated by two authors (S.K. and K.W.), who were independently blinded to the clinical data. The number of immunoreactive cells and that of total cells were counted in three different visual fields, at a magnification of x400. The frequencies of immunoreactive cells were defined as expression scores, and subjected to statistical analysis.

### NGS

We followed the strategy as described previously^20,28^. mtDNA was amplified using four primer pairs^59^ (Supplementary Table) that covered the whole mitochondrial genome.

### Analysis of the NGS data

The analysis was performed as described previously^20,28^ and is summarized in Supplementary Fig. S5. Reads mapped to the mitochondrial genome were deposited into the DRA database (DRA005375 and DRA005377). When considering individual polymorphisms, the consensus sequences were built from each library after downsampling, and bases not determined to be single were replaced by N through the use of fatt clean (https://github.com/mkasa/klab). To exclude the possibility that any reported haplotypes were misrecognized as hypermutated mtDNA, reads with four or more C-to-T or G-to-A mutations were individually subjected to a BLASTN search with the nt dataset, and those more closely matched to reported sequences were excluded.

### Cell culture

W12 cells were cultured for maintenance, or induced to differentiate, as described previously in^27^ and^35,36^, respectively. Immortalized keratinocyte was established, maintained, and induced to differentiate as previously described^37^. NHEK were purchased from Promo Cell (C-12006, Heidelberg, Germany), and maintained in Keratinocyte Growth Medium 2 (D-39006, Heidelberg, Germany). To induce differentiation, NHEK with a passage number of less than two were cultivated with 1.2 mM CaCl_2_ for up to 10 days. siRNA was purchased from Invitrogen (siA3A; HSS-153372-74, negative control; 12935-200 and 12935-400). Lipofectamine RNAiMAX (Thermo Fisher, 13778150) was used for transfection, according to the maker’s protocol. Lentiviral shA3A and shA3B vectors were obtained from Sigma (TRCN0000049958 for shA3A, TRCN0000140546 and TRCN0000157469 for shA3B), and control shRNA vectors from Addgene (17920 for shSCR^60^, and 12273 for siGFP^61^). For preparation of viral supernatants, the lentiviral vectors were co-transfected with helper vectors, pMD2.G (Addgene12259) and psPAX2 (Addgene12260), in 293FT cells (Thermo Fisher, R70007), utilizing CalPhos™ Mammalian Transfection Kit (Clonetech). The supernatants were applied to W12 cells and selected by puromycin.

### Western blotting

Western blotting was performed as previously described^20,27,54,58,62^. The concentration of the lysates was determined by TaKaRa BCA Protein Assay Kit (T9300A), following the manufacture’s protocol. A3A and A3B were blotted by either a rabbit serum that reacted to both A3A and A3B^20^. Other antibodies used in this study were as follows: rabbit anti-keratin 10 (K10) (sab4501656, Sigma), rabbit anti-GAPDH (G9545, Sigma), horseradish peroxidase (HRP)-conjugated anti-rabbit IgG (GE Healthcare, Little Chalfont, UK), mouse anti-HA (Invivogen, San Diego, California), and anti-mouse IgG–HRP (GE Healthcare).

Subcellular fractionation of W12 cells was performed using a Mitochondria Isolation Kit for Cultured Cells (Thermo Fisher, 89874) following the manufacturer’s instructions. After the removal of nuclei and unlysed cells, supernatants were spun down at 3000 × *g* for 15 minutes at 4 °C to harvest mitochondria with minimum contamination of lysosomes or peroxisomes. Anti-HSP-60 (GeneTex, Irvine, California, GTX110089) and β-tubulin (GeneTex, GTX101279) antibodies were used to validate the purification of the mitochondrial and cytosolic fractions, respectively.

### Expression vectors and transfection

pmax GFP was obtained from Lonza (Basal, Switzerland), and pmax A3A was constructed by inserting an A3A open reading frame (NM_145699) with EcoRI/XhoI ends into an AgeI/XhoI arm of pmax GFP. A HA-A3B expression vector was obtained from NIH AIDS Reagent Program (Cat# 11090)^63^. Plasmids were transfected into W12 cells using Fugene 6 (Promega), according to the manufacture’s instruction.

### Statistical analysis

Statistical analyses were performed using GraphPad Prism (GraphPad Software, La Jolla, California). Two-tailed unpaired *t*-tests, Pearson’s chi-squared tests, and Mann-Whitney U-tests were used for the RT-qPCR analysis, mutation analyses, and IHC, respectively. Differences between experimental groups with *P* values < 0.05 were considered statistically significant. In all the graphs presented for this study, the error bars indicate the standard deviation, calculated from duplicated samples^63^.

## Electronic supplementary material

Supplementary Figures

## References

[1] Chen D, Zhan H (2009). Study on the mutations in the D-loop region of mitochondrial DNA in cervical carcinoma. Journal of cancer research and clinical oncology.

[2] Sharma H, Singh A, Sharma C, Jain SK, Singh N (2005). Mutations in the mitochondrial DNA D-loop region are frequent in cervical cancer. Cancer cell international.

[3] Zhai K, Chang L, Zhang Q, Liu B, Wu Y (2011). Mitochondrial C150T polymorphism increases the risk of cervical cancer and HPV infection. Mitochondrion.

[4] Lin JC, Wang CC, Jiang RS, Wang WY, Liu SA (2015). Impact of somatic mutations in the D-loop of mitochondrial DNA on the survival of oral squamous cell carcinoma patients. Plos one.

[5] Prior SL (2006). Mitochondrial DNA mutations in oral squamous cell carcinoma. Carcinogenesis.

[6] Sabharwal SS, Schumacker PT (2014). Mitochondrial ROS in cancer: initiators, amplifiers or an Achilles’ heel?. Nat Rev Cancer.

[7] Wallace DC (2012). Mitochondria and cancer. Nat Rev Cancer.

[8] Ishikawa K (2008). ROS-generating mitochondrial DNA mutations can regulate tumor cell metastasis. Science.

[9] Shidara Y (2005). Positive contribution of pathogenic mutations in the mitochondrial genome to the promotion of cancer by prevention from apoptosis. Cancer research.

[10] Petros JA (2005). mtDNA mutations increase tumorigenicity in prostate cancer. Proceedings of the National Academy of Sciences of the United States of America.

[11] Koshikawa N, Akimoto M, Hayashi JI, Nagase H, Takenaga K (2017). Association of predicted pathogenic mutations in mitochondrial ND genes with distant metastasis in NSCLC and colon cancer. Scientific reports.

[12] Klaunig JE, Kamendulis LM, Hocevar BA (2010). Oxidative stress and oxidative damage in carcinogenesis. Toxicologic pathology.

[13] Kabekkodu SP (2014). Mitochondrial DNA variation analysis in cervical cancer. Mitochondrion.

[14] Goila-Gaur R, Strebel K (2008). HIV-1 Vif, APOBEC, and intrinsic immunity. Retrovirology.

[15] Harris RS, Liddament MT (2004). Retroviral restriction by APOBEC proteins. Nat Rev Immunol.

[16] Malim MH (2009). APOBEC proteins and intrinsic resistance to HIV-1 infection. Philos Trans R Soc Lond B Biol Sci.

[17] Vieira VC, Soares MA (2013). The role of cytidine deaminases on innate immune responses against human viral infections. BioMed research international.

[18] Burns MB, Temiz NA, Harris RS (2013). Evidence for APOBEC3B mutagenesis in multiple human cancers. Nature genetics.

[19] Roberts SA (2013). An APOBEC cytidine deaminase mutagenesis pattern is widespread in human cancers. Nature genetics.

[20] Kondo S (2017). APOBEC3A associates with human papillomavirus genome integration in oropharyngeal cancers. Oncogene.

[21] Suspene R (2011). Somatic hypermutation of human mitochondrial and nuclear DNA by APOBEC3 cytidine deaminases, a pathway for DNA catabolism. Proceedings of the National Academy of Sciences of the United States of America.

[22] Ohba K (2014). *In vivo* and *in vitro* studies suggest a possible involvement of HPV infection in the early stage of breast carcinogenesis via APOBEC3B induction. Plos one.

[23] Landry S, Narvaiza I, Linfesty DC, Weitzman MD (2011). APOBEC3A can activate the DNA damage response and cause cell-cycle arrest. EMBO reports.

[24] Mussil B (2013). Human APOBEC3A isoforms translocate to the nucleus and induce DNA double strand breaks leading to cell stress and death. Plos one.

[25] Fliss MS (2000). Facile detection of mitochondrial DNA mutations in tumors and bodily fluids. Science.

[26] Chatterjee A, Mambo E, Sidransky D (2006). Mitochondrial DNA mutations in human cancer. Oncogene.

[27] Wang Z (2014). APOBEC3 Deaminases Induce Hypermutation in Human Papillomavirus 16 DNA upon Beta Interferon Stimulation. J Virol.

[28] Wakae K (2015). Detection of hypermutated human papillomavirus type 16 genome by Next-Generation Sequencing. Virology.

[29] Vieira, V. C. *et al*. Human papillomavirus E6 triggers upregulation of the antiviral and cancer genomic DNA deaminase APOBEC3B. *mBio* **5** (2014). 10.1128/mBio.02234-14 PMC4278539 25538195

[30] Warren CJ (2015). APOBEC3A functions as a restriction factor of human papillomavirus. J Virol.

[31] Henry M (2009). Genetic editing of HBV DNA by monodomain human APOBEC3 cytidine deaminases and the recombinant nature of APOBEC3G. Plos one.

[32] Stenglein MD, Burns MB, Li M, Lengyel J, Harris RS (2010). APOBEC3 proteins mediate the clearance of foreign DNA from human cells. Nature structural & molecular biology.

[33] Vartanian JP, Guetard D, Henry M, Wain-Hobson S (2008). Evidence for editing of human papillomavirus DNA by APOBEC3 in benign and precancerous lesions. Science.

[34] Hultquist JF (2011). Human and rhesus APOBEC3D, APOBEC3F, APOBEC3G, and APOBEC3H demonstrate a conserved capacity to restrict Vif-deficient HIV-1. J Virol.

[35] Flores ER, Lambert PF (1997). Evidence for a switch in the mode of human papillomavirus type 16 DNA replication during the viral life cycle. J Virol.

[36] Koffa MD, Graham SV, Takagaki Y, Manley JL, Clements JB (2000). The human papillomavirus type 16 negative regulatory RNA element interacts with three proteins that act at different posttranscriptional levels. Proceedings of the National Academy of Sciences of the United States of America.

[37] Egawa N (2012). The E1 protein of human papillomavirus type 16 is dispensable for maintenance replication of the viral genome. J Virol.

[38] Hamanaka RB (2013). Mitochondrial reactive oxygen species promote epidermal differentiation and hair follicle development. Science signaling.

[39] Bause AS, Matsui MS, Haigis MC (2013). The protein deacetylase SIRT3 prevents oxidative stress-induced keratinocyte differentiation. J Biol Chem.

[40] Lackey L, Law EK, Brown WL, Harris RS (2013). Subcellular localization of the APOBEC3 proteins during mitosis and implications for genomic DNA deamination. Cell Cycle.

[41] Allalunis-Turner J, Ma I, Hanson J, Pearcey RG (2006). mtDNA mutations in invasive cervix tumors: a retrospective analysis. Cancer letters.

[42] Mazzei F, Viel A, Bignami M (2013). Role of MUTYH in human cancer. Mutation research.

[43] Sharma S (2015). APOBEC3A cytidine deaminase induces RNA editing in monocytes and macrophages. Nature communications.

[44] Bingol B, Sheng M (2016). Mechanisms of mitophagy: PINK1, Parkin, USP30 and beyond. Free radical biology & medicine.

[45] Stewart JB, Chinnery PF (2015). The dynamics of mitochondrial DNA heteroplasmy: implications for human health and disease. Nature reviews. Genetics.

[46] Holt, I. J. & Reyes, A. Human mitochondrial DNA replication. *Cold Spring Harbor perspectives in biology* **4** (2012). 10.1101/cshperspect.a012971 PMC3504440 23143808

[47] Ju, Y. S. *et al*. Origins and functional consequences of somatic mitochondrial DNA mutations in human cancer. *eLife* **3** (2014). 10.7554/eLife.02935 PMC4371858 25271376

[48] Hoopes JI (2016). APOBEC3A and APOBEC3B Preferentially Deaminate the Lagging Strand Template during DNA Replication. Cell reports.

[49] Chen Wongworawat Y, Filippova M, Williams VM, Filippov V, Duerksen-Hughes PJ (2016). Chronic oxidative stress increases the integration frequency of foreign DNA and human papillomavirus 16 in human keratinocytes. American journal of cancer research.

[50] Henderson S, Chakravarthy A, Su X, Boshoff C, Fenton TR (2014). APOBEC-mediated cytosine deamination links PIK3CA helical domain mutations to human papillomavirus-driven tumor development. Cell reports.

[51] Norman JM (2011). The antiviral factor APOBEC3G enhances the recognition of HIV-infected primary T cells by natural killer cells. Nat Immunol.

[52] Fujinaga Y (1991). Simultaneous detection and typing of genital human papillomavirus DNA using the polymerase chain reaction. The Journal of general virology.

[53] Kukimoto I (2015). Hypermutation in the E2 gene of human papillomavirus type 16 in cervical intraepithelial neoplasia. Journal of medical virology.

[54] Kitamura K (2013). Uracil DNA glycosylase counteracts APOBEC3G-induced hypermutation of hepatitis B viral genomes: excision repair of covalently closed circular DNA. PLoS Pathog.

[55] Liang G (2013). RNA editing of hepatitis B virus transcripts by activation-induced cytidine deaminase. Proceedings of the National Academy of Sciences of the United States of America.

[56] Micallef L (2009). Effects of extracellular calcium on the growth-differentiation switch in immortalized keratinocyte HaCaT cells compared with normal human keratinocytes. Experimental dermatology.

[57] Kovacs D (2012). The eumelanin intermediate 5,6-dihydroxyindole-2-carboxylic acid is a messenger in the cross-talk among epidermal cells. The Journal of investigative dermatology.

[58] Seishima, N. *et al*. Expression and subcellular localization of AID and APOBEC3 in adenoid and palatine tonsils. *Scientific reports* (in press). 10.1038/s41598-017-18732-w PMC5772672 29343743

[59] Dames S (2013). The development of next-generation sequencing assays for the mitochondrial genome and 108 nuclear genes associated with mitochondrial disorders. The Journal of molecular diagnostics: JMD.

[60] Saharia A (2008). Flap endonuclease 1 contributes to telomere stability. Current biology: CB.

[61] Orimo A (2005). Stromal fibroblasts present in invasive human breast carcinomas promote tumor growth and angiogenesis through elevated SDF-1/CXCL12 secretion. Cell.

[62] Ahasan MM (2015). APOBEC3A and 3C decrease human papillomavirus 16 pseudovirion infectivity. Biochemical and biophysical research communications.

[63] Doehle BP, Schafer A, Cullen BR (2005). Human APOBEC3B is a potent inhibitor of HIV-1 infectivity and is resistant to HIV-1 Vif. Virology.

