# Supplementary material for: Keratinocyte differentiation induces APOBEC3A, 3B, and mitochondrial DNA hypermutation
Source: Sci Rep. 2018 Jun 27;8:9745. doi: 10.1038/s41598-018-27930-z (PMC6021414; doi:10.1038/s41598-018-27930-z)
Supplement: Supplementary file 1 — Supplementary Figures [file 41598_2018_27930_MOESM1_ESM.pdf]

Supplementary Information
Keratinocyte differentiation induces APOBEC3A, 3B, and mitochondrial DNA hypermutation

Kousho Wakae, Tomoaki Nishiyama, Satoru Kondo, Takashi Izuka, Lusheng Que, Cong Chen, Kina Kase, Kouichi Kitamura, Md Mohiuddin, Zhe Wang, Md Monjurul Ahasan, Mitsuhiro Nakamura, Hiroshi Fujiwara, Tomokazu Yoshizaki, Kazuyoshi Hosomochi, Atsushi Tajima, Tomomi Nakahara, Tohru Kiyono, and Masamichi Muramatsu

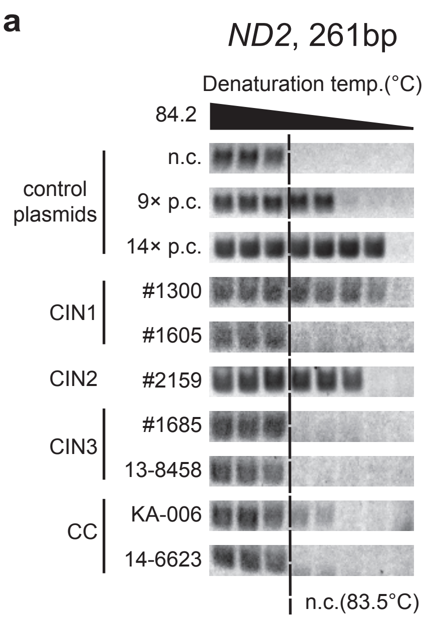

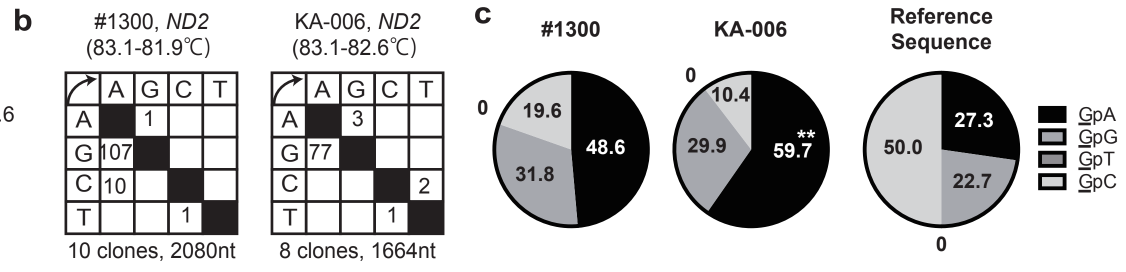

Supplementary Figure S1. *ND2* gene hypermutation in cervical malignancy (Related to Fig.1)
(a) Total DNAs from cervical dysplasia or cancer were subjected to 3D-PCR targeting *ND2*, along with plasmids containing amplicons with the indicated number of C-to-T mutations as control reactions (n.c., 9x p.c., and 14x p.c., corresponds to 0, 9, and 14 C-to-T mutations, respectively). The dotted lines indicate the denaturing temperature 83.5°C, the lowest temperature at which *ND2* was amplified from n.c. The result for seven cervical dysplasia/cancer is representatively shown. (b,c) 3D-PCR products amplified at 83.1°C or lower denaturation temperatures were sequenced and mapped to NC_012920.1. Mutations were summarized in matrices (b), and the result of the 5′-dinucleotide context analysis is shown in (c) (** P < 0.01).

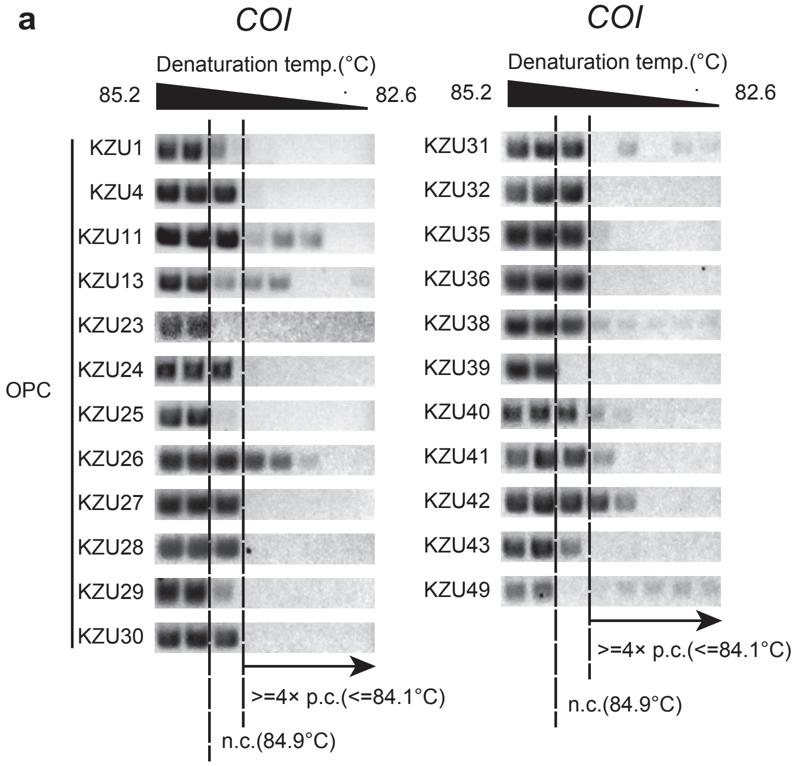

b

HPV		mtDNA hypermutation (<=84.1°C)	% of hypermutation
+	KZU1	-	33.3%(8/24)
	KZU4	-	
	KZU11	+	
	KZU12	-	
	KZU13	+	
	KZU21	-	
	KZU22	+	
	KZU23	-	
	KZU24	-	
	KZU25	-	
	KZU26	+	
	KZU28	-	
	KZU34	-	
	KZU35	-	
	KZU36	-	
	KZU39	-	
	KZU40	-	
	KZU42	+	
	KZU43	-	
	KZU44	-	
	KZU46	+	
	KZU47	+	
	KZU48	+	
	KZU49	-	
-	KZU27	-	33.3%(3/9)
	KZU29	-	
	KZU30	-	
	KZU31	-	
	KZU32	-	
	KZU33	+	
	KZU41	+	
	KZU45	+	

Supplementary Figure S2. HPV infection does not associate with mitochondrial DNA hypermutation (Related to Fig. 1)
(a) 3D-PCR analysis of *COI* from oropharyngeal cancer was performed as in Fig. 1 (b) HPV infection and positivity of COI 3D-PCR analysis were summarized.

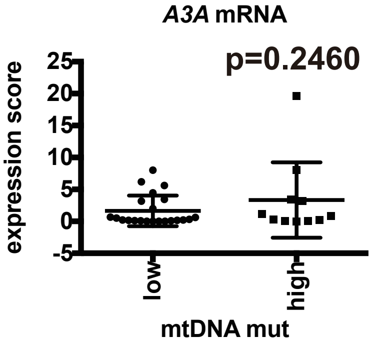

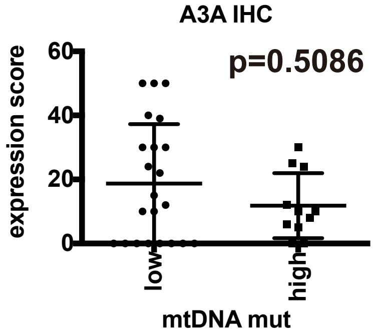

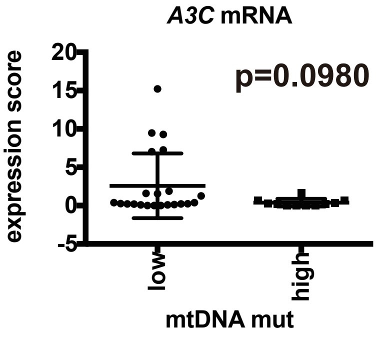

Supplementary Figure S3. Expression level of A3s in OPCs (Related to Fig. 1)

RT-qPCR analysis and Immunohistochemical analysis were performed for OPC specimens to evaluate mRNA and protein levels of A3s. The specimens were divided into low and high mtDNA mutation load accoridng to the lowest denaturing temperature at which *COI* was amplified (higher or lower than 84.1°C). The differences between each group were statistically analyzed by two-tailed student's t-test for RT-qPCR, and Mann-Whitney U-test for immunohistochmistry, respectively.

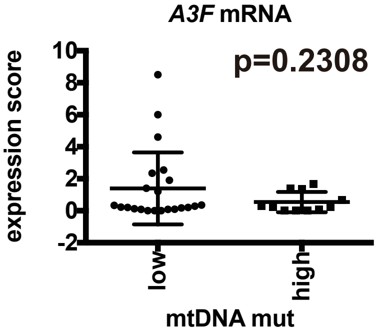

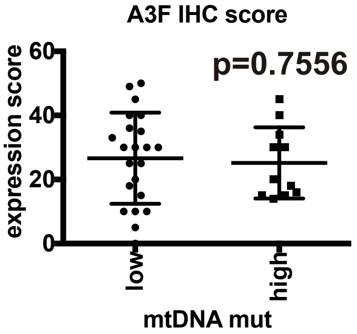

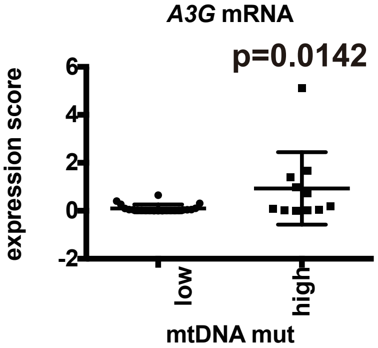

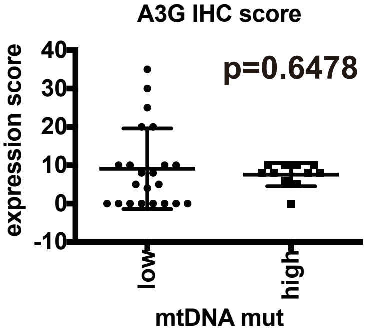

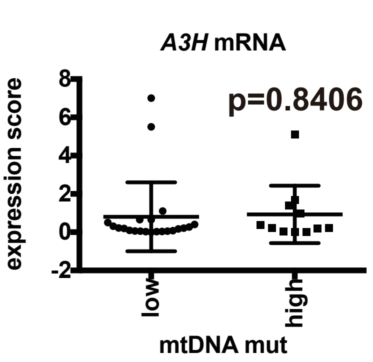

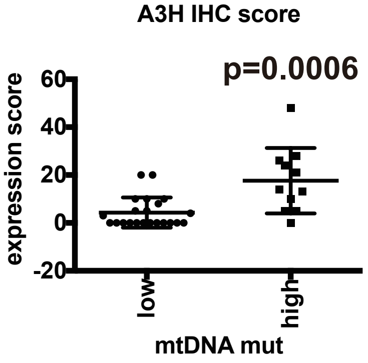

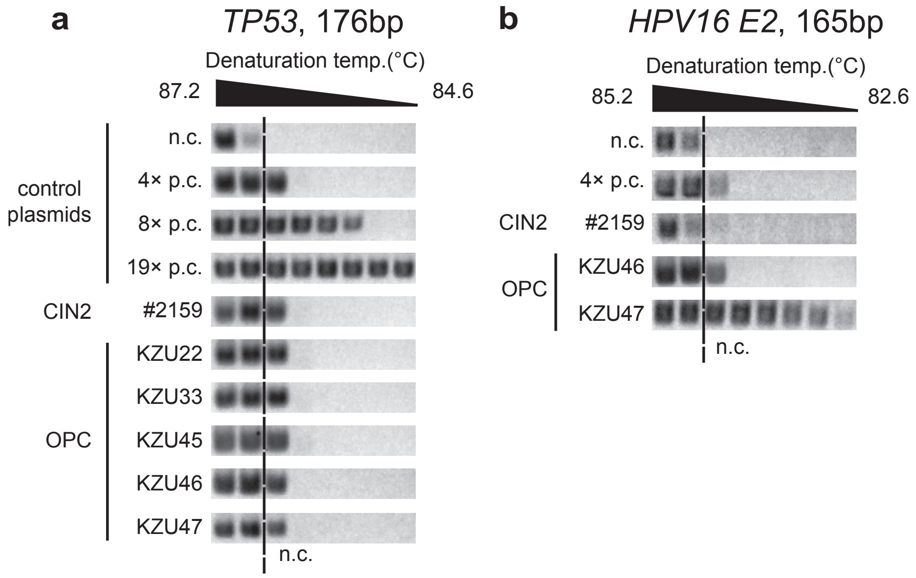

Supplementary Figure S4. 3D-PCR analysis targeting *TP53* and HPV16 *E2* (Related to Fig. 1)
(A, B) CIN2 and OPC samples with mitohondrial DNA hypermutation were subjected to 3D-PCR analysis targeting *TP53* (a) and HPV16 *E2* (b). Plasmids containing the indicated number of C-to-T mutations were simultanously tested.

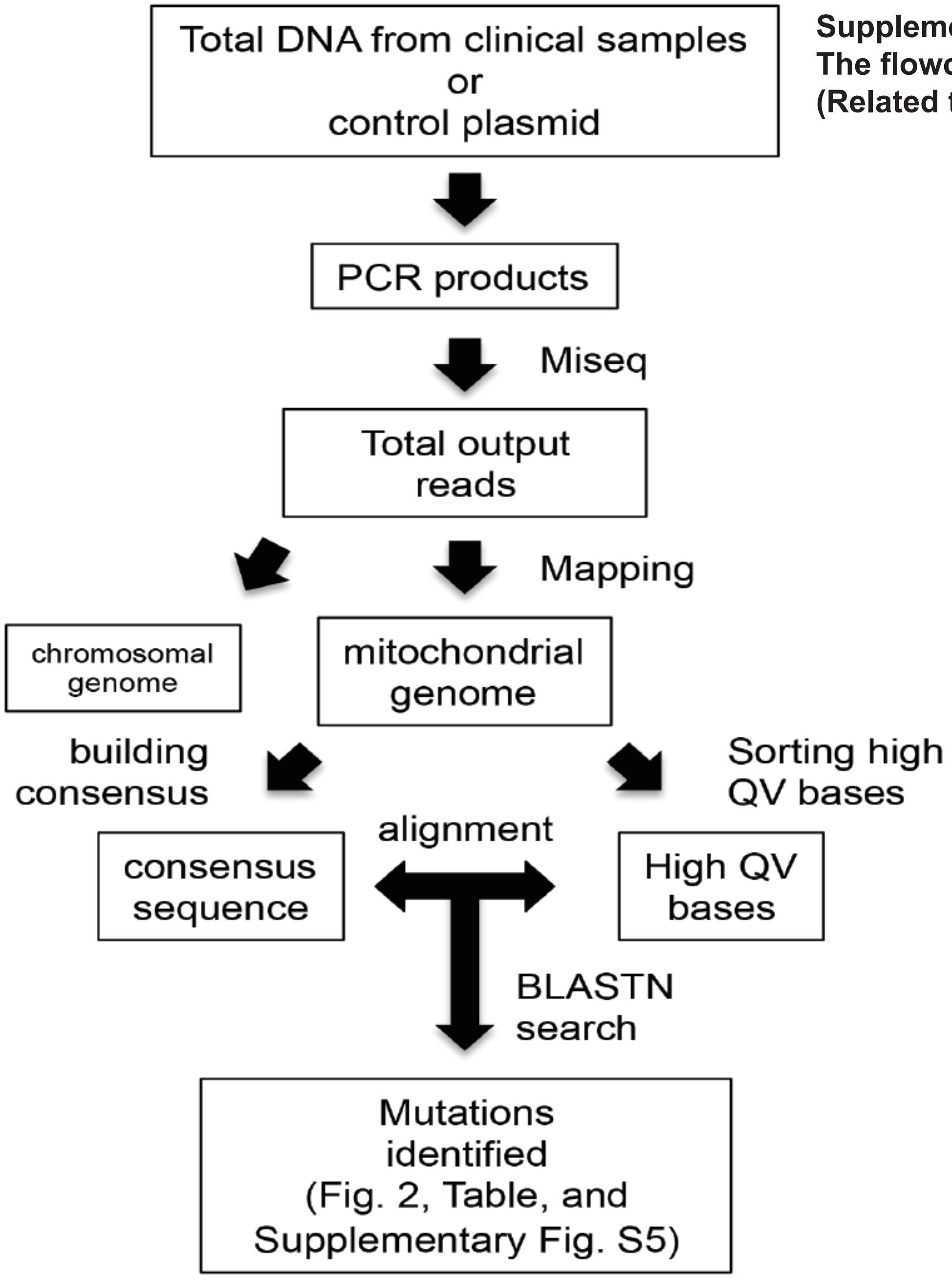

Supplementary Figure S5.
The flowchartof NGS analysis in this study (Related to Fig. 2 and Table)

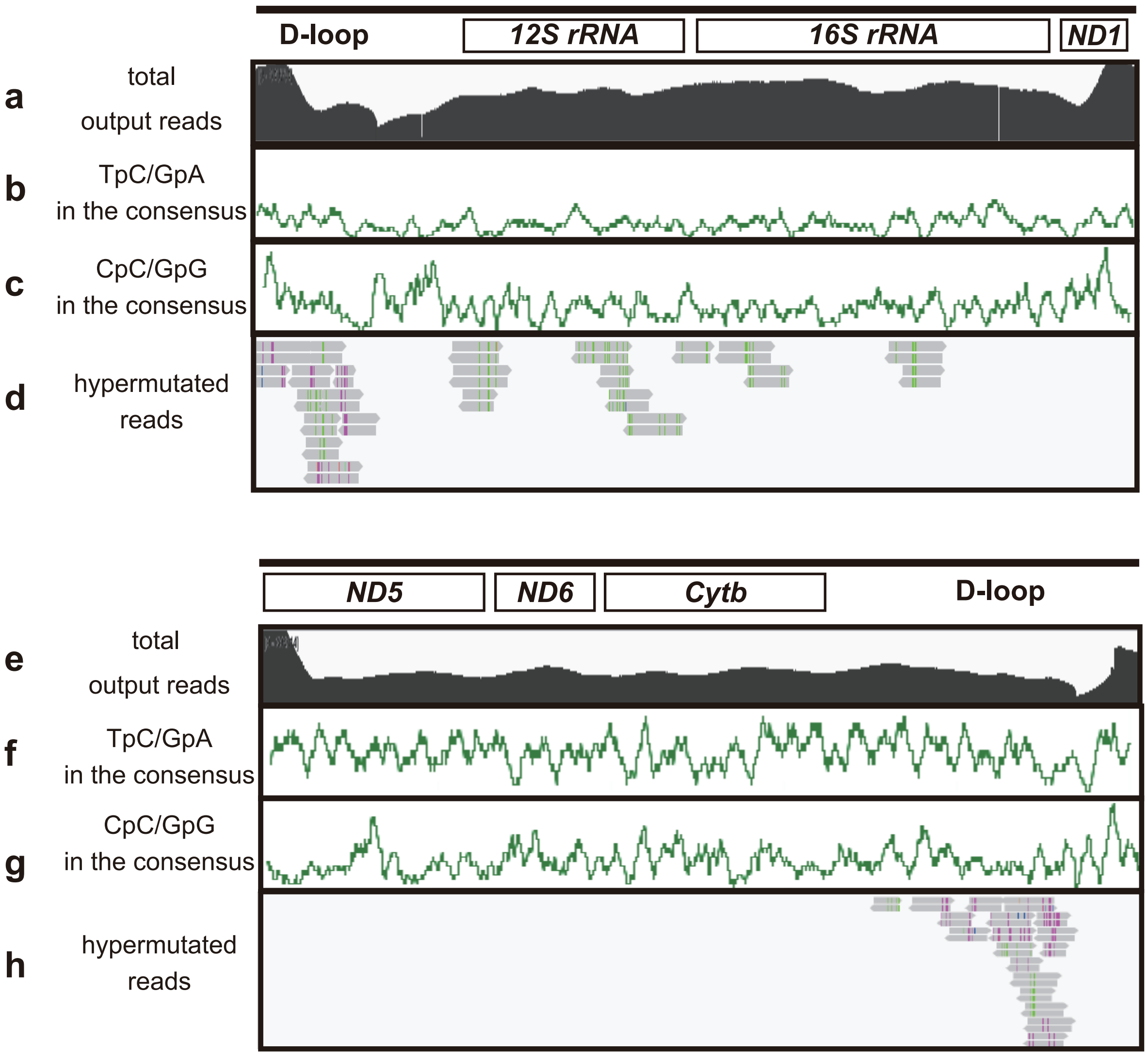

Supplementary Figure S6. Accumulated hypermutation in D-loop in #1300 (Related to Fig. 2)
Reads containing four or more C-to-T/G-to-A mutations in D-loop-*ND1* (a-d) or *ND5-tRNA-F* (e-h) were extracted and mapped to each concensus sequence. (a,e) The coverage and the depth of the total output reads. (b,c,f,g) The frequency of the TpC/GpA (b,f) and CpC/GpG (c,g) dinucleotide contexts per 50-bp of the consensus sequence. (d,h) The reads containing four or more C-to-Tor G-to-A mutations were mapped to each consensus sequence and visualized by Integrative Genomics Viewer. Magenta, green, blue, and orange tics represent the bases substituted by T, A, C, and G, respectively. For reference, D-loop and the genes (*12S rRNA*, *16S rRNA*, *ND1*, *ND5*, *ND6*, and *Cytb*) in the consensus sequence are shown on the top.

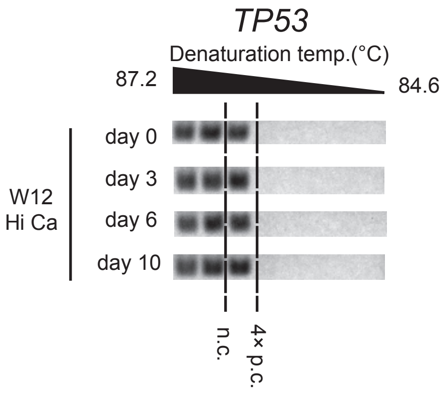

Supplementary Figure S7. 3D-PCR analysis of *TP53* in differentiated W12 cells (Related to Fig. 3)
Total DNAs were extracted from W12 cells cultivated in high extracellular calcium concentration up to day 10. *TP53* 3D-PCR was performed as in Supplementary Fig. S4.

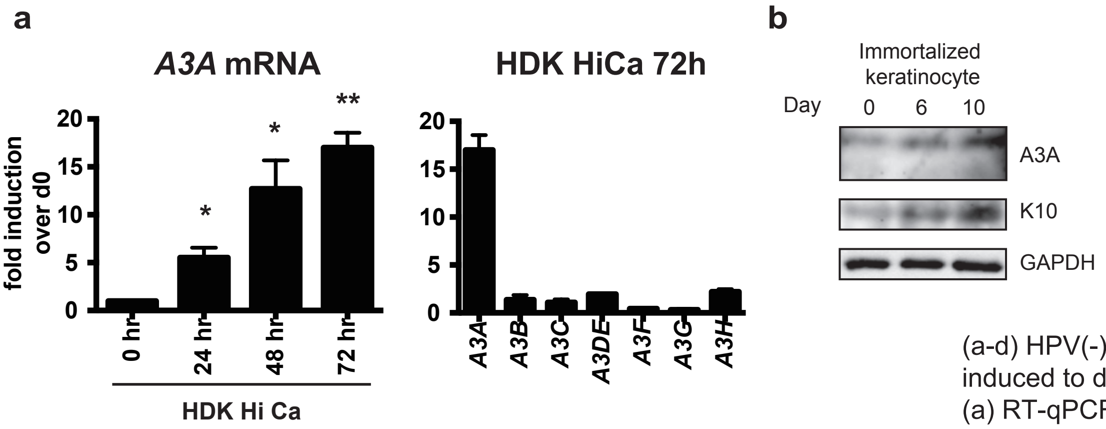

Supplementary Figure S8. mitochondrial DNA hypermutation occurs by differentiation of HPV(-) keratinocytes (Related to Fig.3)

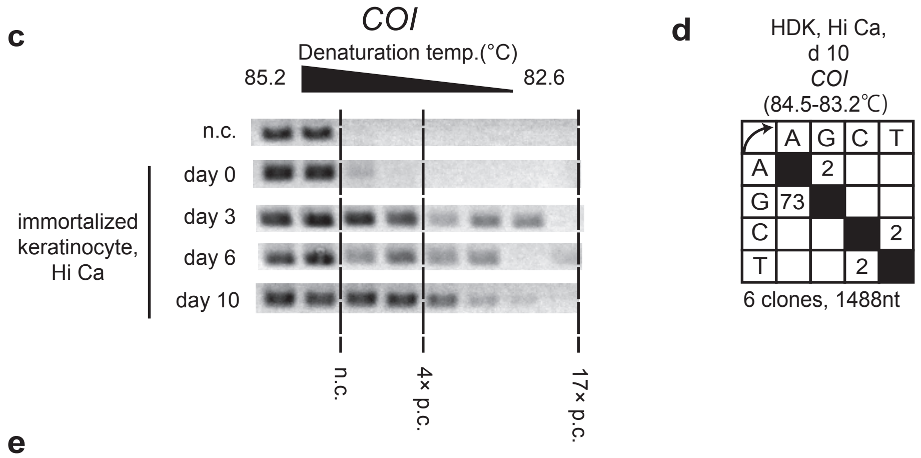

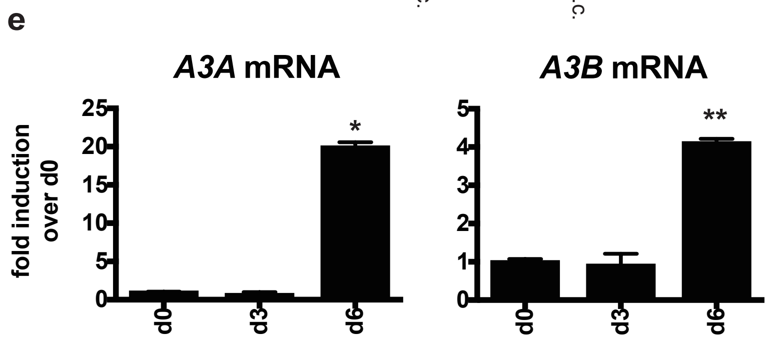

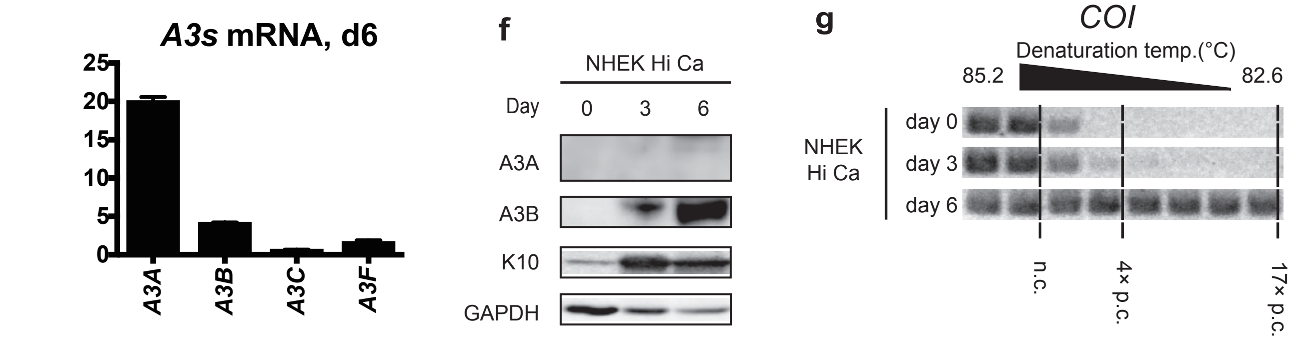

(a-d) HPV(-) immortalized keratinocytes were induced to differentiate in 1.8mM $CaCl_2$. (a) RT-qPCR analysis of *A3* mRNA. Cells were cultivated up to 72 hours. Each mRNA copy numbers of *A3s* was normalized by that of *HPRT*. The mean values and the standard deviations are indicated as bar graphs and error bars, respectively. (b) Whole cell lysates from cells differentiated for 6 or 10 days were subjected to Western Blotting. (c) Total DNAs from differentiated cells were subjected to 3D-PCR targeting *COI*, as in Figs.1 and 3. (d) The amplicons from the denaturing temperature 84.5°C or lower were sequenced and algined with human mtDNA reference sequence (NC_012920.1). (e-g) Normal Human Epidermal Keratinocytes (NHEK) were induced to differentiate in keratinocyte growth medium containing 1.2mM $CaCl_2$, up to 6 days (e) RT-qPCR analysis of *A3* mRNA. (f) Immunobloting of A3A, A3B and K10. 5μg of each lysate was run. (g) 3D-PCR analysis of *COI* gene The P-values were calculated using the unpaired two-tailed student t test . *P<0.05 and **P<0.01.

Supplementary Fig.S9 A3A/A3B silencing in differentiating W12 cells

(a,b) W12 cells were transfected with control siRNA or siA3A at day 0, 3, and 6, and cultivated under high extracellular calcium for 10 days. (a)Total RNA was subjected to RT-qPCR analysis to quantify *A3A* mRNA. (b)Total DNA was subjected to 3D-PCR analysis targeting *COI*. (c-e) Five lentiviral shRNA transfectants were established from W12 cells,(scramble shRNA(shSCR), siGFP,shA3A, and two shA3Bs), and cultivated under high extracellular calcium for 10 days (c,d) RT-qPCR analysis to quantify mRNA levels of *A3A*(c) and *A3B*(d). (e) 3D-PCR analysis targeting *COI.*

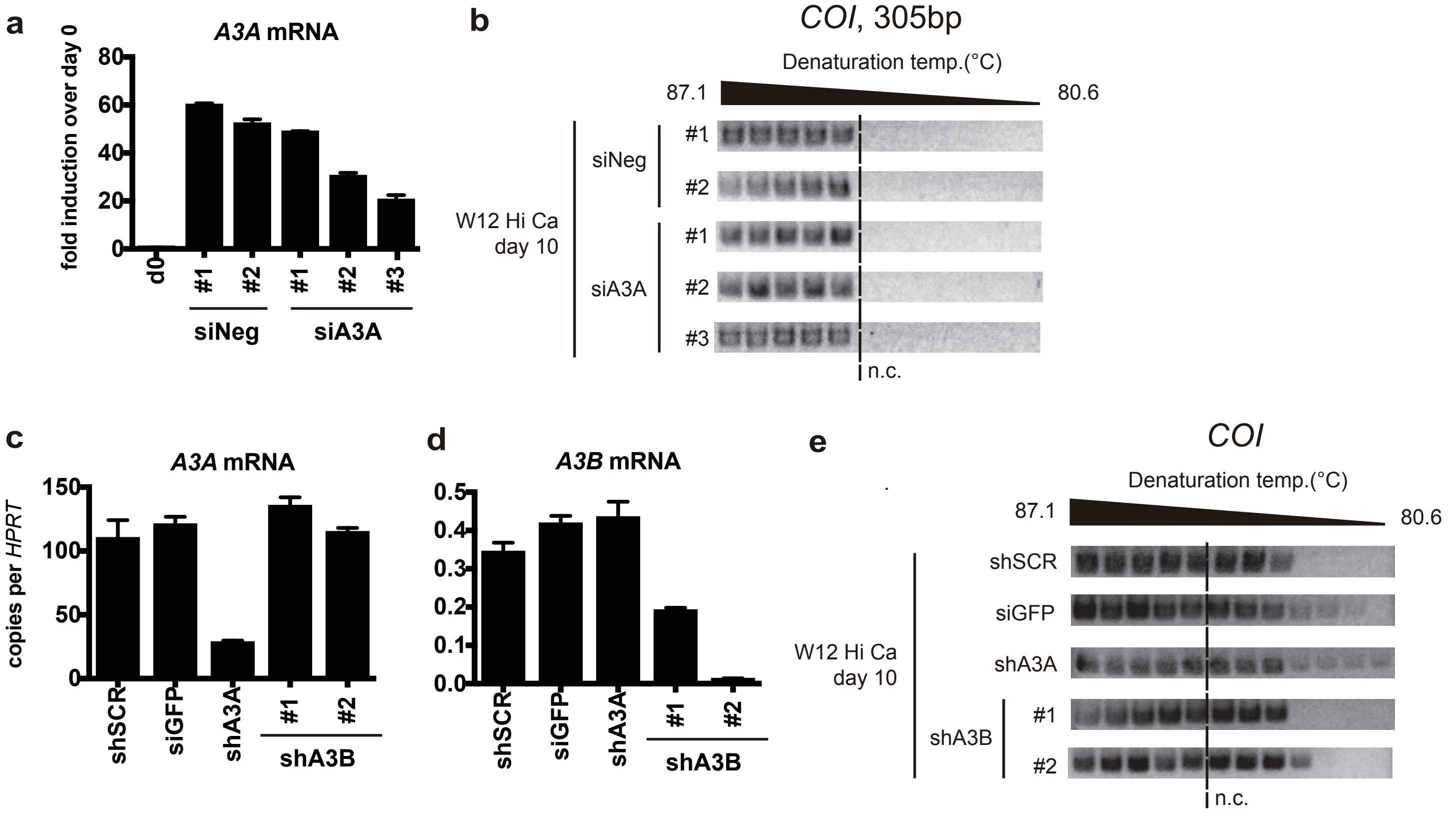

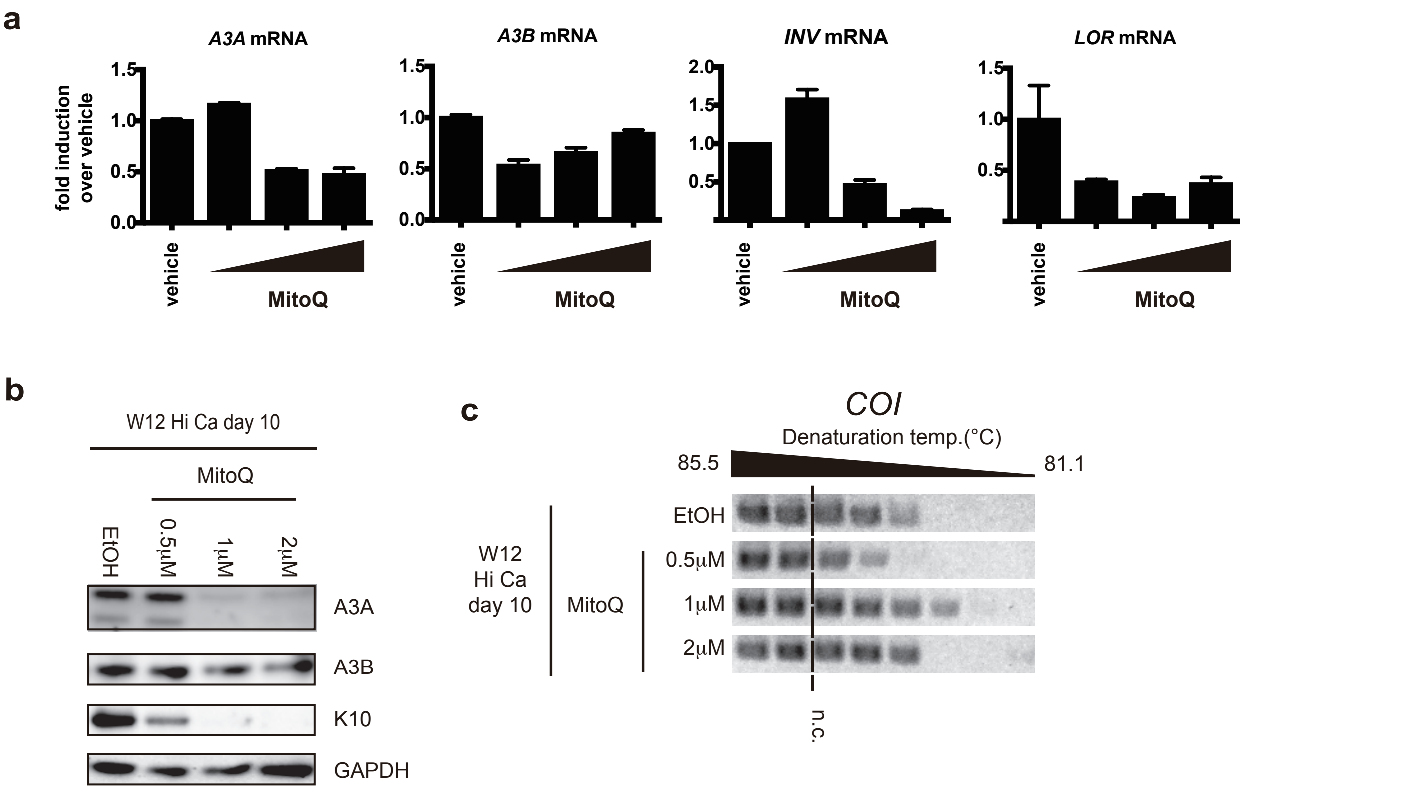

Supplementary Fig. S10 Effect of a ROS antagonist, Mito-Q, on differentiation of W12 cells

W12 cells were induced to differentiate under high extracellular calcium concentration for 10 days in the presence of vehicle (ethanol) or 0.5μM-2μM mitoquinone (MitoQ). The harvested cells were subjected to (a) RT-qPCR, (b) Immunoblotting, and (c) 3D-PCR analysis targeting COI.

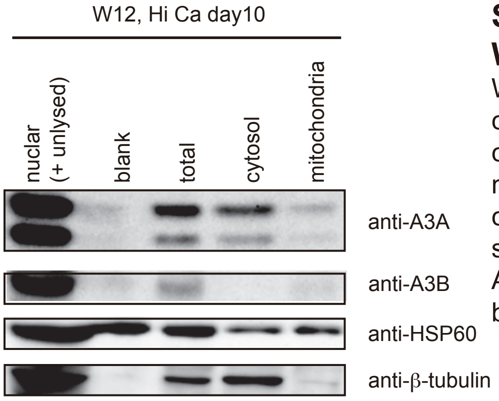

Supplementary Fig. S11 Nuclear fraction of differentiated W12 cells

W12 cells were induced to differentiate under high extracellular calcium concentration for 10 days. Cells were lysed and mildly centrifuged to seperate mitochondrial and cytosolic fraction from nuclear and unlysed fraction. The supernatant was further robustly centrifuged to isolate mitochondrion from cytosol. Each fraction was subjected to westernblot analysis to evaluate protein level of A3A, A3B, HSP60, and β-tubulin. For reference, a scheme of the protocol provided by the manufacturer is indicated below.

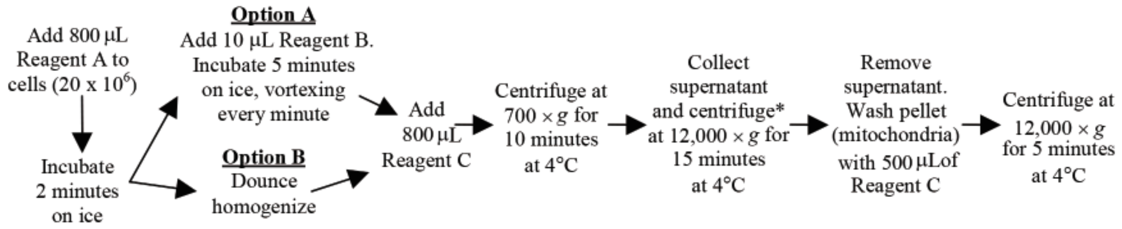

Supplementary Figure S12.
A model for carcinogenesis by A3s, mtDNA mutation, and ROS

Keratinocyte differentiation induces A3A and A3B, that hypermutate mitochondrial DNA as well as HPV and chromosomal DNA [1]. Mitochondrial DNA (mtDNA) mutations leads to decreased mitochondrial function and ROS overproduction, that leads to (i) accumulation of mutations by DNA oxidation [2] (ii) increased DNA damage and integration of HPV genome [3] (iii) increased proliferation and metastatic ability of cancer cells [4,5]. A3s theirselves also mutate host and HPV genome, induces DNA damage and promotes HPV integration [1,6]. Thus, A3s mutate mtDNA to accerate these carciongenic event, via ROS upregulation.

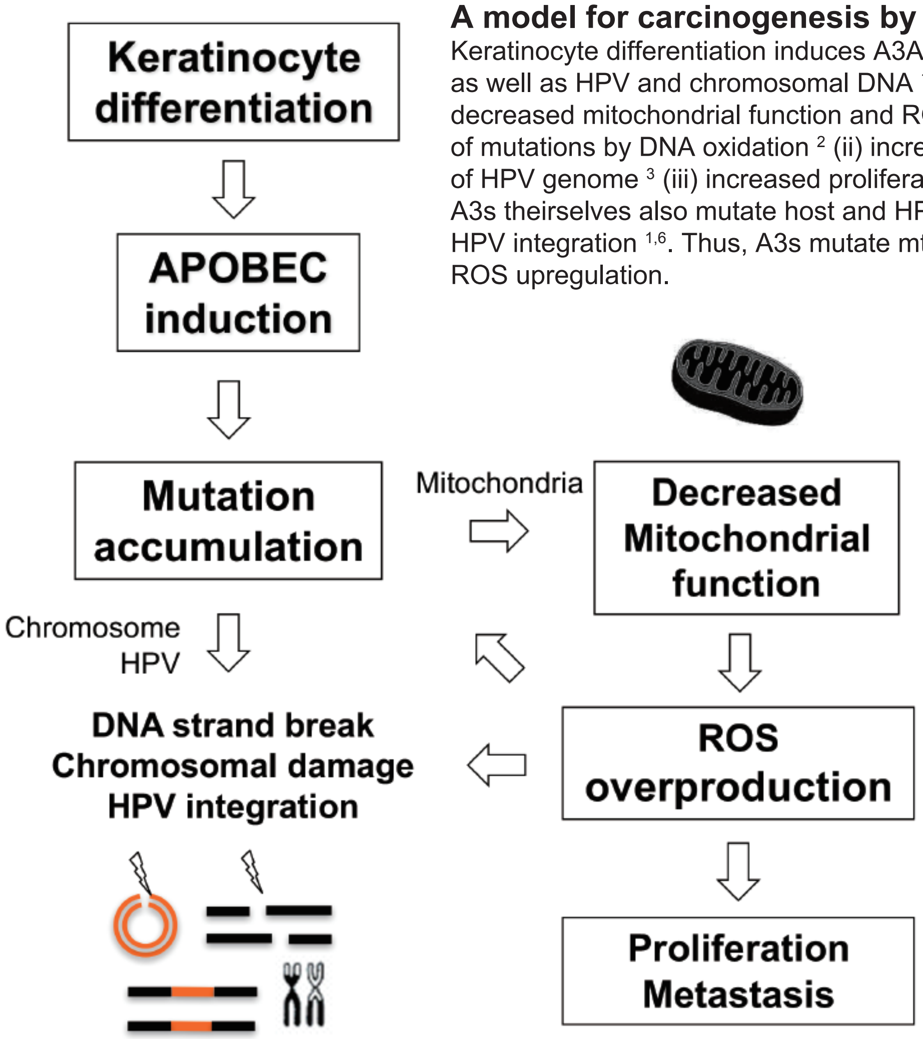

Reference

(1) Kondo, S et al. APOBEC3A associates with human papillomavirus genome integration in oropharyngeal cancers. Oncogene, 36(12):1687-1697. (2017)

(2) Klaunig, J. E., Kamendulis, L. M. & Hocevar, B. A. Oxidative stress and oxidative damage in carcinogenesis.Toxicologic pathology 38, 96-109, (2010).

(3) Chen Wongworawat, Y. et al. Chronic oxidative stress increases the integration frequency of foreign DNA and human papillomavirus 16 in human keratinocytes. American journal of cancer research 6, 764-780 (2016).

(4) Ishikawa, K. et al. ROS-generating mitochondrial DNA mutations can regulate tumor cell metastasis. Science 320, 661-664, (2008).

(5) Petros, J. A. et al. mtDNA mutations increase tumorigenicity in prostate cancer. Proceedings of the National Academy of Sciences of the United States of America 102, 719-724, (2005).

(6) Ohba, K. et al. In vivo and in vitro studies suggest a possible involvement of HPV infection in the early stage of breast carcinogenesis via APOBEC3B induction. PloS one 9, e97787, (2014).

Supplementary Figure S13.

The original images of main figures

The original images of Figs. 1a (a), 3a (b), 3c (c), 4a (d), 4b (e), 5a (f), 5b (g), and 5c (h),respectively.

a

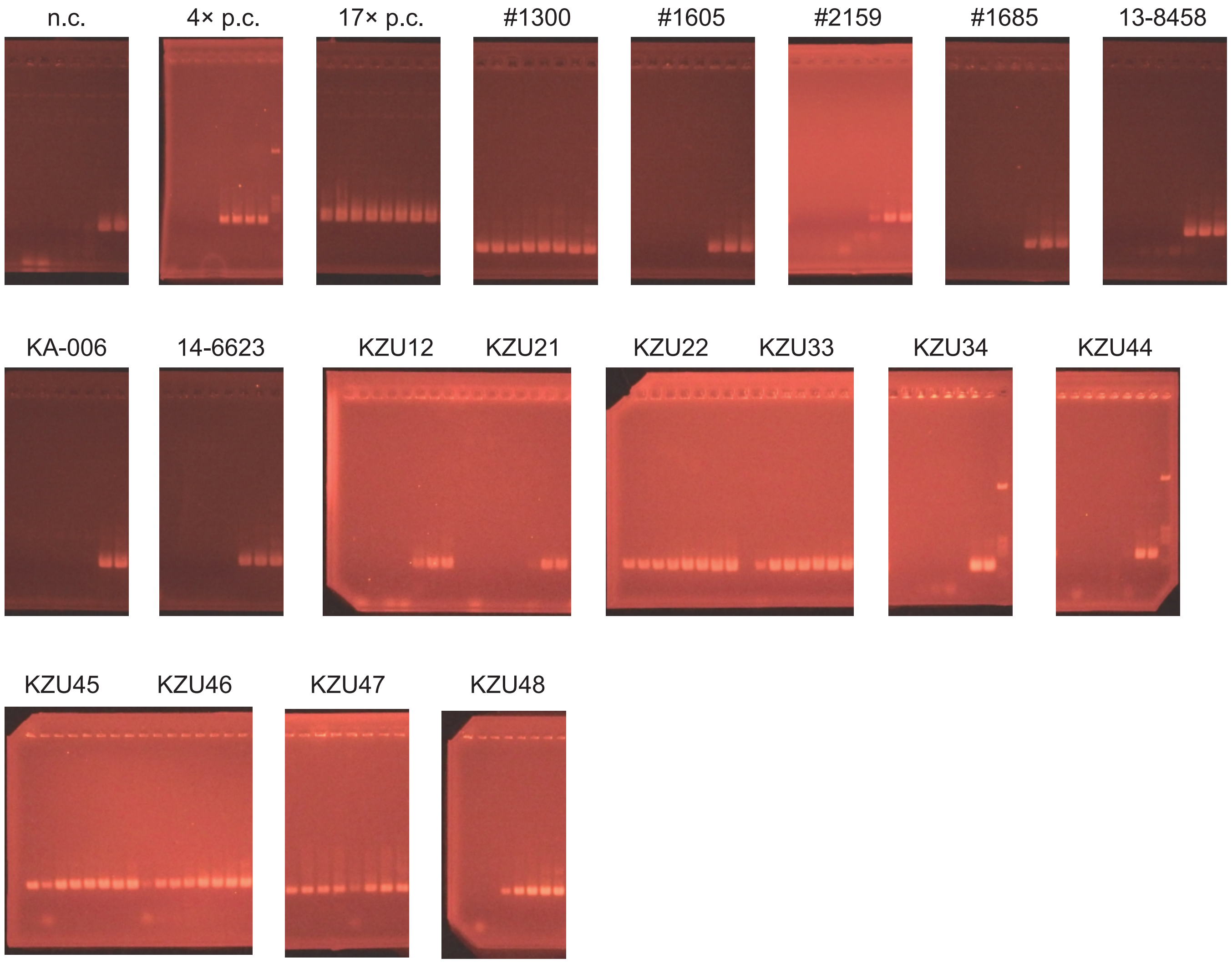

b

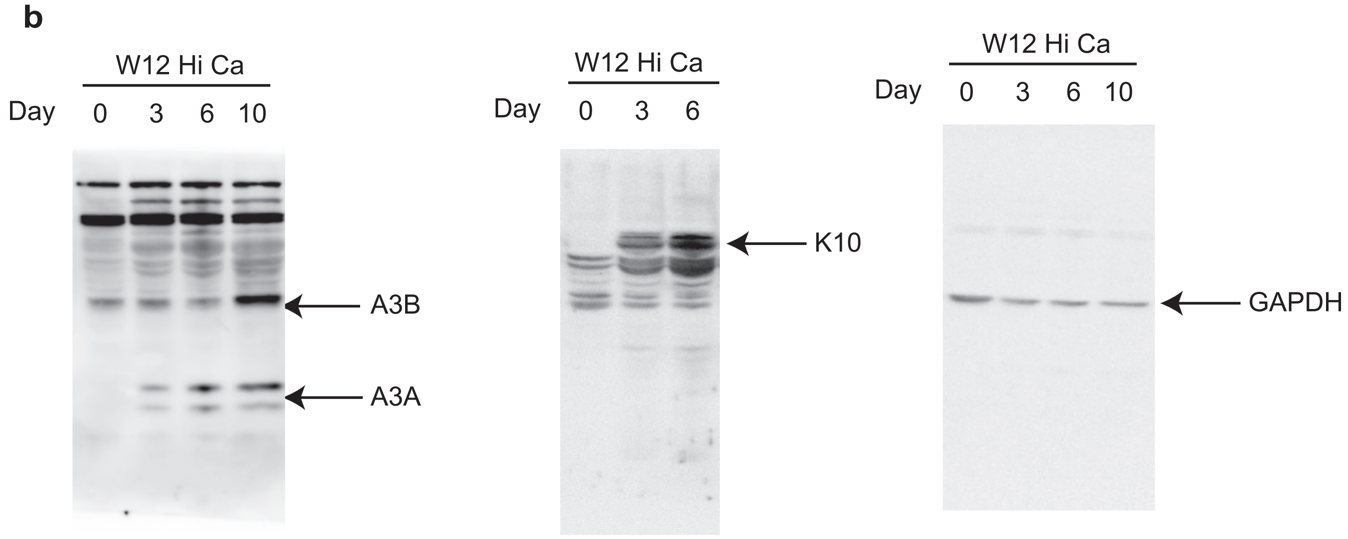

c

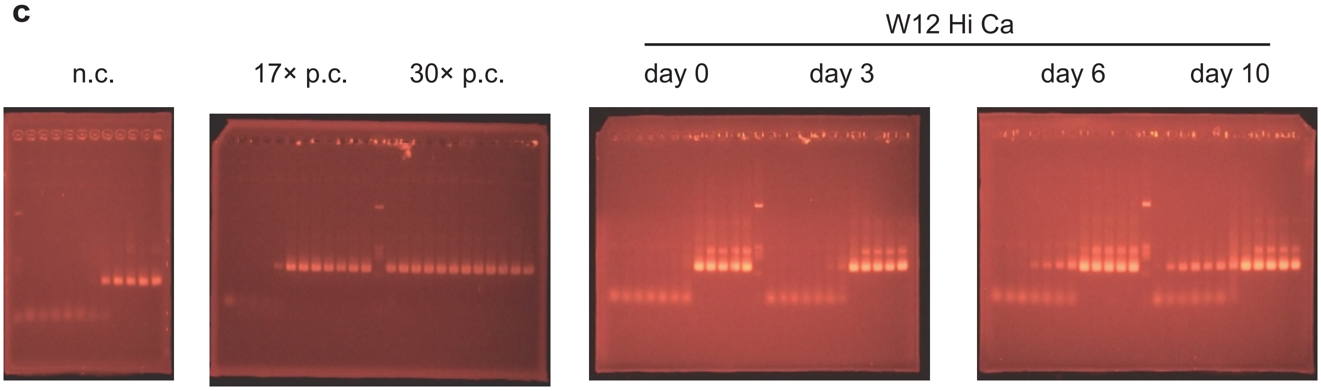
W12 Hi Ca
n.c.
17× p.c.
30× p.c.
day 0
day 3
day 6
day 10

d

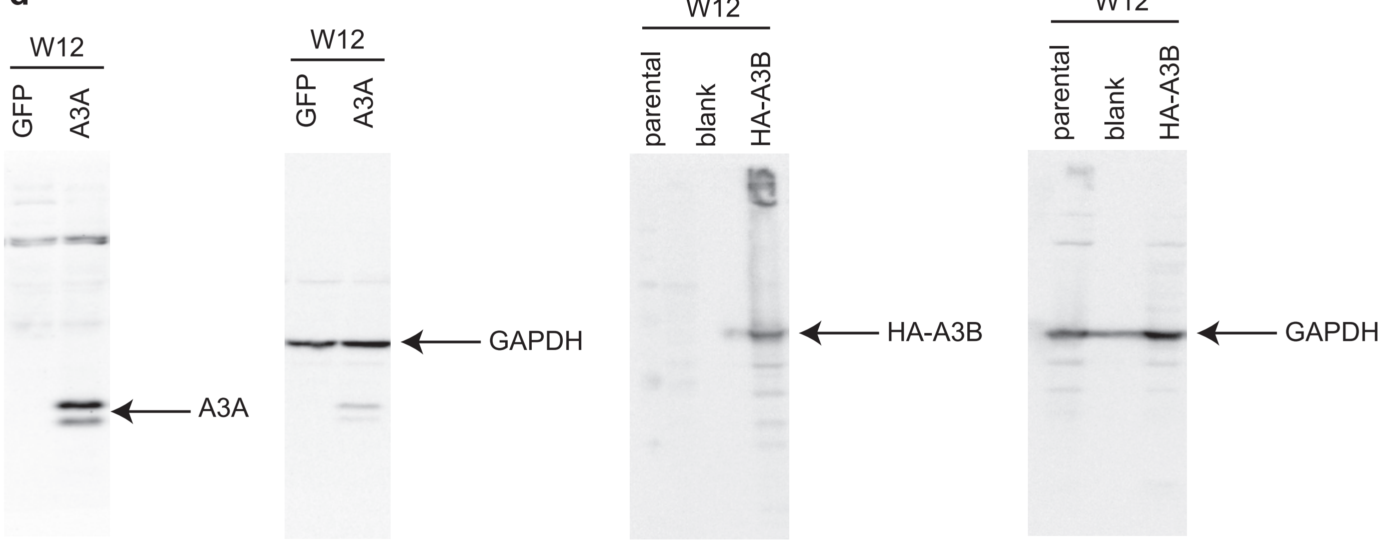
W12
GFP
A3A
A3A
W12
GFP
A3A
GAPDH
W12
parental
blank
HA-A3B
HA-A3B
W12
parental
blank
HA-A3B
GAPDH

e

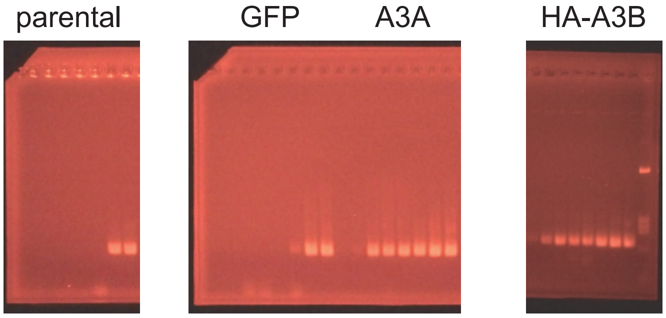
parental
GFP
A3A
HA-A3B

f

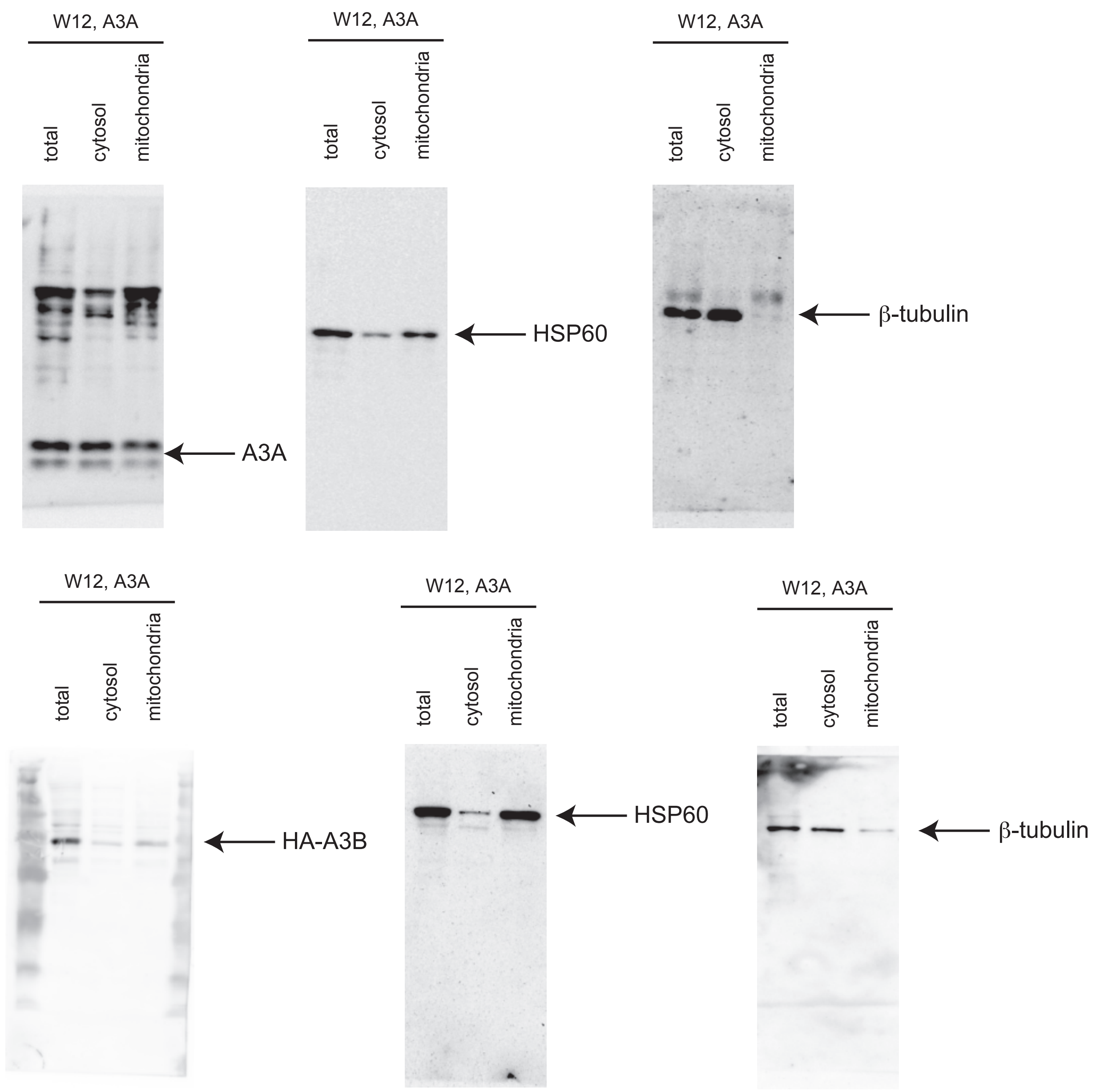

W12, A3A
total
cytosol
mitochondria
A3A
W12, A3A
total
cytosol
mitochondria
HSP60
W12, A3A
total
cytosol
mitochondria
β-tubulin
W12, A3A
total
cytosol
mitochondria
HA-A3B
W12, A3A
total
cytosol
mitochondria
HSP60
W12, A3A
total
cytosol
mitochondria
β-tubulin

g

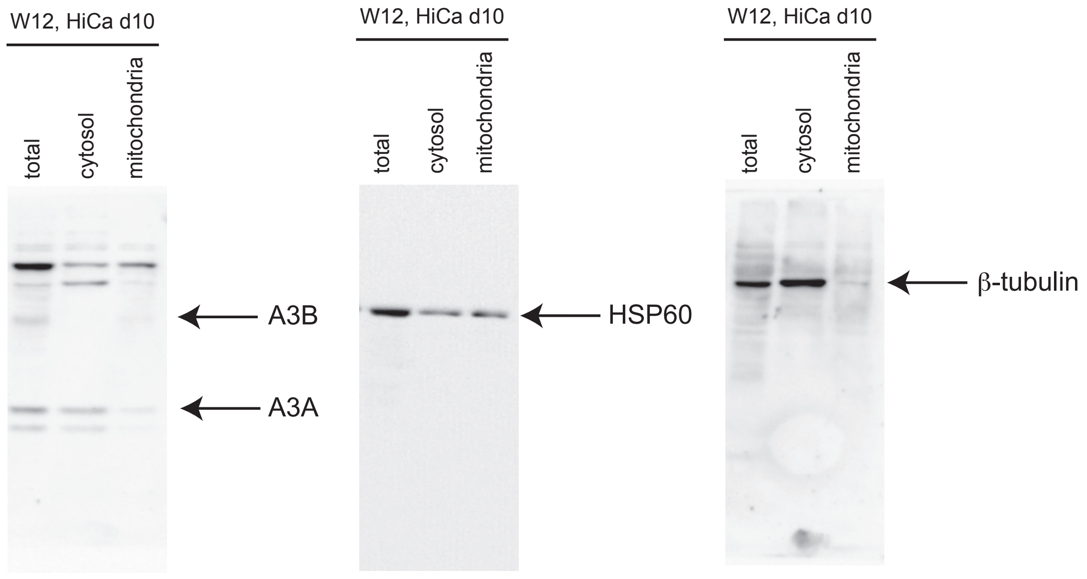

W12, HiCa d10
total
cytosol
mitochondria
A3B
A3A
W12, HiCa d10
total
cytosol
mitochondria
HSP60
W12, HiCa d10
total
cytosol
mitochondria
β-tubulin

h

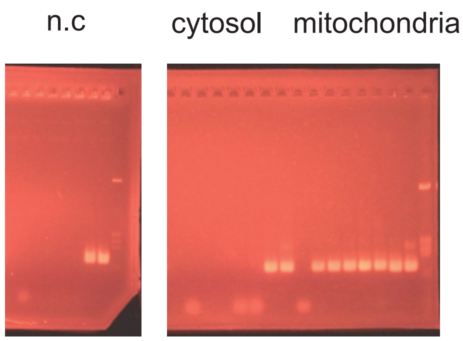

Supplementary Table: Primers used in this study

assay	Figures	target genes	orientation	sequence	reference
HPV16 genotyping, cervical specimens	Fig.1 and Supplementary Fig. S1	HPV16 *E6E7*	F	TGTCAAAAGCCACTGT	Fujinaga et al. J. Gen. Virol. 1991
			R	GAGCTGTCATTTAATTGCTC	
3D-PCR	Figs.1, 3, 4, and 5<br>Supplementary Figs. S2, S8, and S9	*COI,* outer	F	GCGGTTGACTATTCTCTACAAACCACAAA	Suspene et al. Proc. Natl. Acad. Sci. 2011
			R	GGGGGTTTTATATTGATAATTGTTGTGATGAAA	
		COI, inner	F	CGTTATCGTCACAGCCCATGCATTTGTAA	
			R	GAGGAGACACCTGCTAGGTGTAAGGTGAA	
	Supplementary Fig. S1	*ND2,* outer	F	ATAGCAGTTCTACCGTACAACCCTAA	
			R	GGGAGATAGGTAGGAGTAGCGTGGTAA	
		ND2, inner	F	ACCGCATTCCTACTACTCAACTTAAA	
			R	GGTGGAGTAGATTAGGCGTAGGTAGAA	
	Supplementary Figs. S4 and S7	*TP53,* outer	F	GAGCTGGACCTTAGGCTCCAGAAAGGACAA	
			R	GCTGGTGTTGTTGGGCAGTGCTAGGAA	
		TP53, inner	F	TTCTCTTTTCCTATCCTGAGTAGTGGTAA	
			R	AAAGGTGATAAAAGTGAATCTGAGGCATAA	
	Supplementary Fig. S4	HPV16 *E2,* outer	F	ATGGGAAGTTCATGCGGGTGGTCA	Kondo et al. Oncogene 2016
			R	TGGGTGTAGTGTTACTATTACAGTTAAT	Wang et al. J.Virol 2014
		HPV16 *E2*, inner	F	TCCTGAAATTATTAGGCAGCACTT	Kukimoto et al. J. Med. Virol. 2015
			R	CGTCCTTTGTGTGAGCTGTTAAAT	Wakae et al. Virology 2015
NGS	Fig.2<br>Supplementary Figs. S5 and S6	D-loop–*ND1*	F	ACATAGCACATTACAGTCAAATCCCTTCTCGTCCC	Dames et al. The journal of Molecular Diagnostics 2013
			R	TGAGATTGTTTGGGCTACTGCTCGCAGTGC	
		ND1–ATP6	F	TACTCAATCCTCTGATCAGGGTGAGCATCAAACTC	
			R	GCTTGGATTAAGGCGACAGCGATTTCTAGGATAGT	
		ATP6–ND5	F	TCATTTTTATTGCCACAACTAACCTCCTCGGACTC	
			R	GTTGGCATCTGCTCGGGCGT	This study
		ND5–tRNA-F	F	AGCCACAACCCAAACAACCCAGC	
			R	TTTATGGGGTGATGTGAGCC	Dames et al. The journal of Molecular Diagnostics 2013
RT-qPCR	Figs.1 and 3<br>Supplementary Figs. S3, S8, S9 and S10	*A3A*	F	ATGGCATTGGAAGGCATAAG	Liang et al. Proc. Natl. Acad. Sci. 2013<br>Kitamura et al. Plos Pathogens 2013<br>Wang et al. J. Virol 2014<br>Wakae et al. Virology 2015
			R	CAAAGAAGGAACCAGGTCCA	
		A3B	F	TTCGAGGCCAGGTGTATTTCA	
			R	CAGAGATGGTCAGGGTGACA	
		A3C	F	CAACGATCGGAACGAAACTT	
			R	TATGTCGTCGCAGAACCAAG	
		A3D	F	ACCCAAACGTCAGTCGAATC	
			R	GCTCAGCCAAGAATTTGGTC	
		A3F	F	GAAACACAGTGGAGCGAATG	
			R	GAAATGGGGCTCTGATGAAAG	
		A3G	F	GGTCAGAGGACGGCATGAGA	
			R	GCAGGACCCAGGTGTCATTG	
		A3H	F	CCCGCCTGTACTACCACTGG	
			R	GGGTTGAAGGAAAGCGGTTT	
		HPRT1	F	GCCCTGGCGTCGTGATTAGT	
			R	CGAGCAAGACGTTCAGTCCTGTC	
		involucrin	F	GGGTGGTTATTTATGTTTGGGTGG	Micallef et al. Exp. Dermatol. 2009
			R	GCCAGGTCCAAGACATTCAAC	
		loricrin	F	TCATGATGCTACCCGAGGTTTG	Kovacs et al. J. Invest. Dermatol. 2012
			R	CAGAACTAGATGCAGCCGGAGA	
